# *Epimedium koreanum* Nakai attenuates glucocorticoid-induced osteoporosis by suppressing ferroptosis via the KEAP1/NRF2/SLC7A11/GPX4 axis: comparative pharmacodynamic evaluation of four epimedium species and proteomic and metabolomic analyses of epk

**DOI:** 10.3389/fphar.2026.1948777

**Published:** 2026-10-29

**Authors:** Tian-Yang Xu, Pan Li, Zi-Meng Qi, Jing-Zhen Wu, Shi-Chang Li, Jin-Ling Liang, Jia Liu, Peng Yu

**Affiliations:** 1 Innovation Practice Center, Changchun University of Chinese Medicine, Changchun, China; 2 School of Pharmacy, Changchun University of Chinese Medicine, Changchun, China; 3 Institute of Medicine and Drug Research, Qiqihar Medical University, Qiqihar, China; 4 Scientific Research Department, Changchun University of Chinese Medicine, Changchun, China

**Keywords:** epimedium, glucocorticoid‐induced osteoporosis, ferroptosis, KEAP1/NRF2/SLC7A11/GPX4 axis, proteomics, metabolomics

## Abstract

**Objective:** The pharmacodynamics and underlying mechanisms of Epimedium (Yingyanghuo), a traditional Chinese medicine, were investigated in the context of glucocorticoid-induced osteoporosis (GIOP) management, with particular emphasis on ferroptosis inhibition.**Methods:** A rat model of GIOP was developed through dexamethasone (DEX) administration. Animals received extracts of the four Epimedium species; *Epimedium koreanum* Nakai (Epk) was selected for subsequent investigation owing to its superior preliminary efficacy. Evaluations encompassed bone microstructure, osteoclast activity, serum biomarkers of bone metabolism, oxidative stress, lipid peroxidation and iron accumulation indicators (4-HNE and Fe^2+^), and ferroptosis-associated proteins (KEAP1/NRF2/SLC7A11/GPX4 pathway). Integration of proteomics and metabolomics facilitated the identification of key pathways, followed by experimental validation of ferroptosis modulation.**Results:** Epk treatment mitigated DEX-induced bone loss, improved bone microarchitecture, and suppressed osteoclast activity. Multi-omics approaches uncovered reversal of 368 proteins and 127 metabolites in GIOP rats following Epk administration, with enrichment observed in ferroptosis, glutathione metabolism, and oxidative phosphorylation. Treatment with Epk further reduced the elevated femoral 4-HNE and Fe^2+^ levels in GIOP rats. Epk treatment elevated NRF2, HO-1, SLC7A11, and GPX4 and reduced KEAP1 and P53 in bone tissue, consistent with suppression of ferroptosis-related signaling.**Conclusion:** Epimedium, with Epk as the prominent representative, confers substantial protection against GIOP, with effects associated with modulation of the KEAP1/NRF2/SLC7A11/GPX4 pathway and suppression of ferroptosis-related markers in bone tissue, thereby providing a basis for further investigation of its clinical potential in GIOP management.

## 1 Introduction

A metabolic bone disease, osteoporosis (OP) manifests through diminished bone mass, compromised microarchitecture, and heightened skeletal fragility (Kanis et al., 1994; Genant et al., 1999; Aibar-Almazán et al., 2022; He et al., 2025). Globally, its incidence continues to rise, making it one of the foremost diseases affecting elderly health (Johnell and Kanis, 2006; K et al., 2025). Against the backdrop of accelerating population aging, osteoporosis together with fracture-related complications has evolved into a pressing public health issue (Compston et al., 2019; Dong et al., 2023). Patients’ quality of life undergoes marked deterioration, while healthcare resources and socioeconomic systems confront an ever-expanding burden. Glucocorticoids (GCs) are widely used clinically to treat allergic and autoimmune disorders and cancer because of their anti-inflammatory and immunosuppressive properties (Almon et al., 2004; Overman et al., 2013; Zheng et al., 2024a). However, long-term GC administration adversely affects the skeletal system, resulting in accelerated bone deterioration, gradual reduction in bone mineral density, and increased vulnerability to fractures, commonly referred to as glucocorticoid-induced osteoporosis (GIOP) (Adami and Saag, 2019; Humphrey et al., 2023; Ochiai et al., 2024). At the molecular level, GCs exert dual regulatory effects on bone metabolism: negatively modulating osteoblast function to limit new bone matrix synthesis while positively regulating osteoclast-mediated bone resorption. This dual imbalance ultimately triggers a negative bone remodeling equilibrium, manifesting as progressive bone density loss and degenerative changes inbone microarchitecture, the core pathological featuresof glucocorticoid-induced osteoporosis (Mohanty et al., 2025; Shi et al., 2025; Song et al., 2025). GIOP exhibits high prevalence, rapid progression, and increased fracture risk, severely impacting patients’ quality of life and prognosis.

Ferroptosis, an iron-dependent form of regulated cell death characterized by iron accumulation, lipid peroxidation, and glutathione peroxidase 4 (GPX4) suppression, has emerged as a critical pathophysiological mechanism in diverse diseases including neurodegenerative disorders, malignancies, and ischemia-reperfusion injury (Lee et al., 2020; Zheng et al., 2024b; Li et al., 2025; Zheng and Conrad, 2025). The metabolic interplay between iron homeostasis and bone remodeling is particularly noteworthy, as iron imbalance disrupts skeletal homeostasis through Fenton reaction-mediated ROS generation, leading to osteoblast dysfunction and exacerbated osteoclast activity (Balogh et al., 2018; Jing et al., 2023; Wu et al., 2024; Xiao et al., 2025). The KEAP1-NRF2 signaling pathway serves as the central regulatory node in cellular antioxidant defense. Under physiological conditions, KEAP1 promotes continuous ubiquitination and proteasomal degradation of NRF2, maintaining low basal expression levels (M et al., 2018; Wang et al., 2022b; Deng et al., 2024). In response to oxidative stress, reactive cysteine residues in KEAP1 are oxidized, leading to KEAP1 inactivation, increased stabilization of NRF2, and its subsequent translocation into the nucleus, thereby activating the expression of downstream antioxidant genessuch as SLC7A11 and GPX4 (Dong et al., 2021; Wang et al., 2022a; Xu et al., 2024). The SLC7A11/GPX4 axis constitutes a pivotal ferroptosis checkpoint. SLC7A11 mediates cystine uptake for glutathione biosynthesis, while GPX4 utilizes glutathione to reduce phospholipid hydroperoxides, thereby preventing membrane lipid peroxidation. Dysregulation of this pathway creates permissive conditions for ferroptotic cell death through compromised redox homeostasis (Koppula et al., 2021; Lei et al., 2021; H et al., 2025; Li et al., 2025). Although the precise mechanisms of ferroptosis in GIOP remain unclear and therapeutic strategies targeting this pathway are still exploratory, the KEAP1/NRF2/SLC7A11/GPX4 signaling axis serves as a central hub coordinating cellular antioxidant defense and ferroptosis regulation, offering important directions for understanding GIOP pathogenesis and developing interventions.

Current pharmacological treatments for osteoporosis, including antiresorptive and anabolic agents (Ruan et al., 2023), are often associated with significant side effects, especially with long-term use (Chai et al., 2024). Referred to in Chinese as “Yinyanghuo”, Epimedium belongs to the Berberidaceae family and plays a significant role in traditional medicinal practices, in which the dried aerial parts are primarily used (Wang et al., 2025). According to the Chinese Pharmacopoeia (2025 edition), it can be classified into *Epimedium brevicornu* Maxim (Epb), *Epimedium* *sagittatum* (Sieb. et Zucc.) Maxim (Eps), *Epimedium koreanum* Nakai (Epk), and *Epimedium pubescens* Maxim (Epp). In TCM, Epimedium is widely used to treat symptoms such as kidney yang deficiency, soreness in the lower back and knees, and weakness in the tendons and bones. Modern pharmacological research indicates that Epimedium possesses multiple pharmacological effects, including anti-inflammatory, antioxidant, anti-fatigue, and immune-enhancing properties. Recent studies have revealed that Epimedium extracts can improve the bone microenvironment by counteracting oxidative stress and suppressing inflammatory responses (Jiao et al., 2021; Zhu et al., 2021; Hq et al., 2024; Tong et al., 2025). However, systematic research on whether it modulates GIOP through regulation of the ferroptosis pathway remains lacking. In addition, the existence of multiple Epimedium species with differing pharmacological substance bases may influence anti-osteoporotic efficacy. This presents a crucial entry point for further investigation into its mechanisms of action. Furthermore, beyond Epimedium, naturally occurring bioactive compounds, such as the polyphenols characteristic of the Mediterranean diet, have been increasingly recognized as safe adjuncts for the prevention and treatment of osteoporosis (Perrone et al., 2025), and situating TCM-derived findings within this broader context of diet-derived bioactive compounds may help identify complementary therapeutic strategies.

This study investigated the pathological mechanisms of GIOP and the interventional effects of Epimedium from the perspective of ferroptosis. By integrating multi-omics technologies with molecular biological validation, we systematically elucidated the scientific basis of Epimedium in the prevention and treatment of osteoporosis. These findings provide a scientific foundation for the clinical application of Epimedium in GIOP management and support its further development as an osteoporosis therapy.

## 2 Materials and methods

### 2.1 Preparation and authentication of epimedium extracts

The four Epimedium raw materials (Epb, Eps, Epk, Epp; origins and batch numbers in Chemicals and antibodies) were authenticated by Associate Professor Guangzhi Cai (Changchun University of Chinese Medicine) as the dried leaves of *Epimedium brevicornu* Maxim., *Epimedium sagittatum* (Sieb. et Zucc.) Maxim., *Epimedium koreanum* Nakai and *Epimedium pubescens* Maxim. (Berberidaceae). Authentication was performed after purchase, and no voucher specimens were deposited in a herbarium. The dried leaves were extracted with 70% ethanol by ultrasonication, and the four extracts were chemically standardized as detailed in the Supplementary Methods (S1). Briefly, the powdered leaves (passed through a No. 3 sieve) were extracted with 70% ethanol (approximately 1:5, w/v) by ultrasonication (400 W, 50 kHz) for 1 h, and the ethanol was removed by rotary evaporation. The total flavonol glycosides (epimedin A, epimedin B, epimedin C and icariin) in each extract were quantified by HPLC, and the HPLC fingerprints and content determination results are shown in Supplementary Figure S1 and Supplementary Table S2 of the Supplementary Material. Extracts were stored at −20 °C until use.

### 2.2 Chemicals and antibodies

Epb (Batch No.: TA220201001) was purchased from Lixian County, Gansu Province; Eps (Batch No.: TA220250001) was purchased from Fengdu County, Chongqing; Epk (Batch No.: TA220571001) was purchased from Tonghua City, Jilin Province; Epp (Batch No.: TA220252001) was purchased from Dianjiang County, Chongqing. Dexamethasone Sodium Phosphate Injection(Batch No.: H41020055)was purchased from Zhengzhou Zhuofeng PharmaceuticalCo., Ltd.; alendronate sodium tablets (Batch No.: J20130085) were purchased from Savio Industrial S. r.L (Italy); paraformaldehyde fixative was purchased from Shanghai McLean Biochemical TechnologyCo., Ltd.; hematoxylin and eosin dyes were purchased from Semmerfels TechnologyCo., Ltd.; osteocalcin (OCN, LOT: NO.231123129R), osteoprotegerin (OPG, LOT: 231123125R), alkaline phosphatase (ALP, LOT: 231123123R), type I collagen carboxyl C-terminal cross-linked peptide (CTX-1, LOT: 231123148R) were purchased from Jiangsu Enzyme-free Industrial Co., Ltd. RANK (1039986-2, Abcam, 1:1000); RANKL(23408-1-AP, Proteintech, 1:500, RRID:AB_2879273); OPG (DF6824, Affinity, 1:2000, RRID:AB_2838784); RUNX2 (20700-1-AP, Proteintech, 1:1000, RRID:AB_2722783); GPX4 (30388-1-AP, Proteintech, 1:500, RRID:AB_3086304); NRF2 (WL02135, Wanlei biology, 1:1000, RRID:AB_2847838); HO-1 (10701-1-AP, Proteintech, 1:1000, RRID:AB_2118685); SLC7A11 (CY7046, Abways, 1:1000); KEAP1 (10503-2-AP, Proteintech, 1:2000, RRID:AB_2132625); FTH1 (11682-1-AP, Proteintech, 1:1000, RRID:AB_3669131); P53 (10442-1-AP, Proteintech, 1:5000, RRID:AB_2206609); ACSL4 (22401-2-AP, Proteintech, 1:2000, RRID:AB_2832995); β-actin (66009-1-lg, Proteintech, 1:20000, RRID:AB_2782959); GAPDH (60004-1-lg, Proteintech, 1:50000, RRID:AB_2737588)antibodies were used.

### 2.3 GIOP model establishment and treatment with *epimedium*

SPF-grade male Sprague Dawley (SD) ratsweighing 200–220 g were procuredfrom Beijing Spefo Biotechnology Co., Ltd. (Beijing, China) and kept under standard conditions in an SPF-grade animal experimental facilityat 20 °C ± 5 °C and 55% ± 5% relative humidity. To ensure a dry and hygienic environment, clean filtered water and normal chow were changed once a day and bedding was changed every other day. The rats were weighed twice a week, and experiments began after 7 days of acclimatization. All animal procedures were performed in strict accordance with the ethics committee for the use of experimental animals at Changchun University of Chinese Medicine(Approval No. 2023558).

A total of 70 male SD rats were randomly allocated into seven groups (n = 10 per group) using a computer-generated randomization table: Control, Model, alendronate sodium (ALN), and four groups receiving different botanical origins of Epimedium herbs (Epb, Eps, Epk, Epp). Investigators responsible for outcome assessment (micro-CT, histology, ELISA, and biochemical assays) were blinded to group allocation. After 1 week of acclimatization, osteoporosis was induced in all groups except the Control group by intramuscular injection of dexamethasone sodium phosphate (2.5 mg/kg) twice weekly for 9 weeks, while the Control group received equivalent physiological saline. During the 14-week intervention period, the ALN group was administered alendronate sodium (1.05 mg/kg·d) via oral gavage, the Epimedium groups were administered their respective extracts by oral gavage at a dose equivalent to 1050 mg/kg·d of the crude herbal material, converted from the maximum daily dose of Epimedium recorded in the Chinese Pharmacopoeia (2020 edition) (6–10 g/day; calculated at 10 g/day for a 60-kg adult) using body-surface-area-based interspecies scaling with a human-to-rat conversion factor of 6.3 (10 g ÷ 60 kg × 6.3 = 1050 mg/kg), and the Control and Model groups were given distilled water. Body weight was measured twice weekly, and all animals had free access to food and water. At the end of the 14-week treatment, samples were collected after an 18-h fast.

### 2.4 Micro-CT

The right femurs were isolated from the surrounding soft tissueand fixed in 4% paraformaldehyde for 72 h. Following removal of the right femur sample from the paraformaldehyde solution, it was scanned using a QuantumGX micro-CT imaging system (PerkinElmer, Inc., Waltham, MA, USA) with an isotropic voxel size of 45 µm and an X-ray source operated at 90 kV/88 µA. A defined region of interest (ROI) in the distal femoral metaphysis was analyzed using a fixed thresholding procedure, and bone mineral density was calibrated against a standard hydroxyapatite phantom. Three-dimensional image reconstruction and quantitative morphometric analysis were performed using the vendor-supplied QuantumGX software (PerkinElmer, Inc.). The relative bone volume(BV/TV, %), bone surface area/tissue volume ratio(BS/TV, 1/mm), bone surface area/bone volume ratio(BS/BV, 1/mm), trabecular thickness(Tb.Th, mm), trabecular separation(Tb.Sp, mm), trabecular number(Tb.N, 1/mm), and bone mineral density(BMD, g/cm^3^)of each femur were collected.

### 2.5 Bone morphology and histopathological analysis

The right femur samples used for micro-computed tomography scanning were decalcified in 0.5 M ethylenediaminetetraacetic acid (EDTA, pH 8.0) for 3 months, with the solution changed once a month. The resulting paraffin-embedded samples were cut longitudinally into 5 μm-thick sections for hematoxylin and eosin (H&E) and TRAP staining. TRAP + cells with red staining and a distinct border were identified as osteoclasts. For all histological stains, three non-adjacent sections per animal and five randomly selected fields per section were analyzed; quantification was performed using ImageJ by two independent observers blinded to group allocation. Stained sections were observed under a Nikon Eclipse Ci light microscope equipped with a Nikon DS-U3 imaging system (Nikon, Japan).

For immunohistochemistry (IHC) staining, paraffin sections were incubated in 0.01 M citrate buffer for 4 h at 60 °C, followed by inactivation in H2O2 for 20 min. Next, sections were incubated with primary antibody (OPG, OCN, RUNX2, KEAP1, NRF2, HO-1) overnight at 4 °C. The next day, DAB solution was applied, followed by hematoxylin counterstaining. Quantitative analysis of IHC staining (integrated optical density) was performed using ImageJ on three sections per animal with five fields per section, by observers blinded to group allocation.

Paraffin slices were prepared identically to IHC for use in bone tissue immunofluorescence (IF) staining. Primary antibodies against GPX4, SLC7A11, P53 were added and incubated overnight at 4 °C. After rinsing, sections were incubated for 60 min at room temperature in the dark with goat anti-rabbit Alexa Fluor 488 or goat anti-rat Alexa Fluor 555 secondary antibodies. DAPI was then applied and incubated for 10 min in the dark at room temperature. Images were captured using a fluorescence microscope (Nikon, Japan). Positive staining was quantified using ImageJ (RRID:SCR_003070) by two independent observers blinded to group allocation.

### 2.6 ELISA detection of serum ALP, OCN, OPG, CTX-1, GSH and GPX4 levels

Serum samples were retrieved from −80 °C storage, and the levels of ALP, OCN, OPG, and CTX-1, biomarkers of bone formation and bone resorption, were measured in each group according to the ELISA kit instructions. Serum GSH and GPX4 levels were measured in the same manner.

### 2.7 Measurement of MDA and SOD

MDA and SOD levels in femoral tissue were measured using an MDA assay kit (Beyotime, S0131) and an SOD assay kit (Beyotime, S0101S), respectively, according to the manufacturer’s instructions.

### 2.8 Measurement of GSH/GSSG and NADP+/NADPH

GSH/GSSG and NADP+/NADPH ratios in femoral tissue were determined using the corresponding assay kits (Beyotime, S0053 and S0179, respectively) according to the manufacturer’s instructions.

### 2.9 Measurement of 4-HNE and Fe^2+^

The 4-HNE content in femoral tissue homogenates was determined using a Rat 4-hydroxynonenal ELISA kit (Cusabio Biotech, cat. no. EQ027232RA), and the Fe^2+^content was determined using a ferrous ion content assay kit (Solarbio, cat. no. BC5415) after BCA protein quantification, both performed according to the manufacturers’ instructions.

### 2.10 4D label-free proteomics

#### 2.10.1 Protein extraction

Tissue samples were homogenized in 300 μL of 8 M urea lysis buffer supplemented with protease inhibitors (10% v/v of lysate volume). The homogenates were centrifuged at 14,100 × g for 20 minat 4 °C, and the resulting supernatants were collected. Protein concentration was determined using the Bradford assay with bovine serum albumin as standard. Aliquots were stored at−80 °C for subsequent analysis.

#### 2.10.2 Protein digestion and desalination

Protein extracts (100 μg per sample) were reduced with 200 mM dithiothreitol (DTT) at37 °C for 1 h, followed by alkylation with iodoacetamide. Samples were diluted four-fold with 25 mM ammonium bicarbonate (ABC) buffer and digested with sequencing-grade trypsin (enzyme-to-protein ratio 1:50) at37 °C overnight. Digestion was terminated by acidification with 0.1% formic acid (FA).

Peptide desalting was performed using C18 StageTips. Columns were preconditioned with 100 μL acetonitrile (ACN) and centrifuged at 1,200 rpm for 3 min, followed by equilibration with 0.1% FA. Peptide samples were loaded onto the columns and centrifuged at 1,200 rpm for 3 min. After two washes with 0.1% FA, columns were rinsed with pH 10 aqueous solution. Peptides were eluted with 70% ACN/0.1% FA, and eluates were lyophilized and stored at−80 °C.

#### 2.10.3 LC-MS/MS analysis

Peptide separation was performed on a nano‐liquid chromatography system coupled to a timsTOF HT mass spectrometer equipped with a CaptiveSpray ion source. Mobile phase A consisted of 100% water with 0.1% formic acid (FA), and mobile phase B consisted of 80% acetonitrile (ACN) with 0.1% FA. The lyophilized peptide sample was reconstituted in 10 µL of solvent A and centrifuged at 14,000 × g for 20 min at 4 °C, and the supernatant containing 400 ng of peptides was collected and injected for LC‐MS/MS analysis. Peptides were separated at a flow rate of 500 nL/min using the following gradient: 3.5%–32% solvent B over 17 min, 32%–95% B over 1 min, 95% B for 2 min, 95%–1% B over 1 min, and 1% B for 1 min (total run time, 22 min).

Mass spectrometry data were acquired in data-dependent acquisition (DDA) PASEF mode. Full MS scans were performed over a mass range of m/z 100–1700 at a resolution of 60,000 (at m/z 1222), with an ion accumulation time of 100 ms in the TIMS tunnel. The capillary voltage was set to 1.6 kV, and the ion mobility range was 0.6–1.6 cm^2^/(V). The total cycle time was 1.1 s, comprising 10 PASEF cycles. Raw data were acquired in .d format. Proteomic analysis was performed by Beijing Qinglian Biotech Co., Ltd. (Beijing, China).

#### 2.10.4 Proteomics data analysis

Raw MS files were processed using FragPipe (RRID:SCR_022864) software against the NCBI and UniProt (RRID:SCR_002380) databases. Search parameters included: trypsin digestion with up to 2 missed cleavages; carbamidomethylation of cysteine as fixed modification; oxidation of methionine and protein N-terminal acetylation as variable modifications; precursor mass tolerance of ±15 ppm; and fragment mass tolerance of ±0.02 Da. Protein and peptide identifications were filtered at an FDR of <1%. Label-free quantification intensities were log2-transformed and median-normalized, and only proteins with valid values in at least two-thirds (≥2/3, ≈70%) of samples within at least one group were retained. Three biological replicates per group (n = 3) were analyzed. Differentially expressed proteins (DEPs) were defined as those with |fold change| ≥ 1.2 and P < 0.05 (Student’s t-test).

### 2.11 Untargeted metabolomics

#### 2.11.1 Processing methods of femur sample

Femur samples (100 mg) were pulverized in liquid nitrogen and homogenized with 3 volumes of ice-cold 80% methanol lysis buffer using a tissue homogenizer at 45 Hz for 4 min. The homogenates were sonicated in an ice-water bath for 5 min followed by incubation at−20 °C for 30 min. After centrifugation at 14,000 × g for 30 minat 4 °C, the supernatants were collected and filtered through 0.45-μm membrane filters. Aliquots (20 μL) were transferred to LC-MS vials for analysis. A quality control (QC) sample was prepared by pooling equal volumes of supernatants from all samples.

#### 2.11.2 Q exactive mass spectrometry metabolite analysis conditions

Chromatographic separation was performed using a Thermo Scientific Dionex Ultimate 3000 RSLC system equipped with a Waters ACQUITY UPLC BEH C8 column (1.7 μm, 2.1 × 100 mm). The mobile phase consisted of (A) acetonitrile/water (60:40, v/v) and (B) isopropanol/acetonitrile (90:10, v/v), both containing 0.1% formic acid and 10 mmol/L ammonium formate. The gradient elution was programmed as follows: 2%–70% A (0–5 min), 70%–100% A (5–8 min), 100% A (8–14 min), 100%–2% A (14–16 min), and 2% A (16–20 min) at a flow rate of 0.25 mL/min.

Mass spectrometry analysis was conducted on a Q Exactive mass spectrometer (Thermo Scientific) with a HESI ion source. The ion source parameters were set as follows:spray voltage 2,500 V, and capillary temperature320 °C. Full MS scans were acquiredat a resolution of 70,000 (m/z 100–1,500) with an AGC target of 3 × 10^6^and maximum injection time of 100 ms. Data-dependent MS^2^ acquisition was performed at 17,500 resolution using stepped normalized collision energies (NCE) of 10, 30, and 60 eV,with an AGC target of 1 × 10^5^ and a maximum injection time of 50 ms.

#### 2.11.3 Metabolomics data analysis

Raw data files were processed using Progenesis QI 2.3 software. Peak alignment was performed with retention time (0.2 min) and mass accuracy (5 ppm) tolerances. Peak detection criteria included:coefficient of variation <30%, signal-to-noise ratio >3, and minimum intensity thresholdof 100,000 in sum-ion mode. Differential metabolites were identified using OPLS-DA with a variable importance in projection (VIP) > 1, |fold change| ≥ 1.2, and P < 0.05, and model robustness was evaluated by 200× permutation testing, with the OPLS-DA score plots shown in Supplementary Figure S5 and the R^2^ and Q^2^ values reported in Supplementary Table S3. Metabolite annotations were assigned with identification confidence levels according to the Metabolomics Standards Initiative (MSI) criteria (Level 2, MS/MS-based; or Level 3, putative annotation). Molecular formula prediction was performed using exact mass and fragment ion matching against metabolic databases. Quantified peak areas were normalized and transformed into a data matrix for subsequent statistical analysis. Five biological replicates per group (n = 5) were analyzed.

### 2.12 Western blot (WB)

The left femur of each rat was collected. Bone tissue was ground in liquid nitrogen, and the powdered tissue was transferred into a 1.5-mL EP tube, lysed with EDTA-free RIPA buffer containing protease inhibitors, and homogenized with two grinding beads in a tissue grinder. Total protein was quantified using a BCA protein assay kit (Beyotime Biotechnology, China, P0010S). A total of 30 μg of protein per lane was separated on 8%–12% sodium dodecyl sulfate–polyacrylamide gel electrophoresis (SDS-PAGE) gels and transferred onto polyvinylidene fluoride (PVDF) membranes at 100 V for 90 min. Membranes were blocked with 5% skim milk in Tris-buffered saline containing 0.1% Tween-20 (TBST) for 2 h at room temperature and then incubated with primary antibodies (dilutions listed in the Chemicals and antibodies section) overnightat 4 °C, followed by incubation with HRP-conjugated secondary antibodies for 1 h at room temperature. Bands were visualized using enhanced chemiluminescence and detected with a Tanon-5200 chemiluminescence imaging system (Tanon, Shanghai, China). Densitometric analysis was performed using ImageJ (RRID:SCR_003070), and target protein levels were normalized to β-actin or GAPDH.

### 2.13 RT-qPCR

Total RNA was extracted from femoral tissue homogenates using TRIzol reagent (TaKaRa Bio, Inc., Otsu, Japan). RNA purity was assessed by the A260/A280 ratio (1.8–2.0), and RNA integrity was verified by 1% agarose gel electrophoresis. One microgram of total RNA was reverse-transcribed using the Transgen kit (AT-401). For the reverse transcription-quantitative polymerase chain reaction (RT-qPCR), the pre-designed primers are listed in Table 1. The reference-gene primers (β-actin and GAPDH) are also provided in Table 1. SYBR Green Master Mix (Transgen, AT-601) was prepared according to the kit’s instructions, and 20-μL reactions containing 10 ng cDNA were amplified on a Thermo A7500 instrument with the following cycling conditions: 95 °C for 5 min, followed by 40 cycles of 95 °C for 15 s and 60 °C for 30 s. Primer amplification efficiencies were verified to be within 90%–110%, and each sample was run in triplicate technical replicates. Relative mRNA expression was calculated using the 2^−ΔΔCt^ method.

**TABLE 1:** Primer sequences for RT-qPCR.

Gene name	Sequence
RANK	F: CCACCTGCCTCTGGAAACG R: GACTGACGTACACCACGATG
RANKL	F: AGCCGAGACTACGGCAAGTA R: GCGCTCGAAAGTACAGGAAC
OPG	F: GAATGGTCACTGGGCTGTTT R: CTGGCAGCTTTGCACAATTA
RUNX2	F: GAGCACAAACATGGCTGAGA R: TGGAGATGTTGCTCTGTTCG
NRF2	F: CCACTGCTCCGACTAGCCAT R: AATCAAATCCATGTCCTGCTGGG
ACSL4	F: GACTGGCAGGAAGGTGGTTA R: CCCATGGAGATATTCTGGCCT
KEAP1	F: GATTGACAGCGTGGTCCGAG R: AGGTTGAAGAACTCCTCCTCCC
HO-1	F: GTCCCAGGATTTGTCCGAGG R: GGAGGCCATCACCAGCTTAAA
P53	F: CCCTGAAGACTGGATAACTGTCA R: CGCTGTGGTGGGCAGAATA
SLC7A11	F: GTTCAGACGATTGTCAGACAGAA R: GGCAGATGGCCAAGGATTTG
GPX4	F: CCGGCTACAATGTCAGGTTT R: ACGCAGCCGTTCTTATCAAT
FTH1	F: ACGTCTATCTGTCCATGTCTTGTT R: GAAGATTCGTCCACCTCGCT
β-actin	F: CTGACACCTTCACCATTCCAG R: ATTGCTGACAGGATGCAGAAG
GAPDH	F: AGACAGCCGCATCTTCTTGT R: TGATGGCAACAATGTCCACT

### 2.14 Statistical analysis

Data are expressed as the mean ± standard deviation and were analyzed using GraphPad Prism 10. Comparisons among multiple groups were performed using one-way ANOVA followed by Tukey’s *post hoc* test, and longitudinal body-weight data were additionally analyzed using two-way ANOVA with repeated measures (mixed-effects model) followed by Šídák’s multiple-comparison test. Significance levels were denoted as *P < 0.05, **P < 0.01, ***P < 0.001, and ****P < 0.0001. For Western blot, RT-qPCR, and immunofluorescence analyses, six biological replicates per group (n = 6) were used, whereas body weight, micro-CT, histology, serum ELISA, and biochemical assays used n = 10 per group.

## 3 Results

### 3.1 Epimedium treatment delays bone loss induced by GIOP in rats

The GIOP rat model was successfully established via DEX injection, as evidenced by a significant suppression of body weight gain in the Model group compared to the Control group during weeks 2–9 of the experiment (Figure 1a). Treatments with different extracts of Epimedium effectively reversed this weight loss trend after 14 weeks of continuous intervention (Figure 1d). Organ coefficient analysis indicated that Epimedium amelioratedthe DEX-induced reduction in kidney weight (Figure 1e). Consistent with these findings, two-way repeated-measures ANOVA with Šídák’s multiple-comparison test confirmed that the body weight of the Model group was significantly lower than that of the Control group from week 2 onward, whereas treatment with ALN or each of the four Epimedium extracts significantly improved body weight relative to the Model group from weeks 2–3 onward; the complete set of pairwise comparisons at each week is shown in Supplementary Figure S2.

**FIGURE 1:**
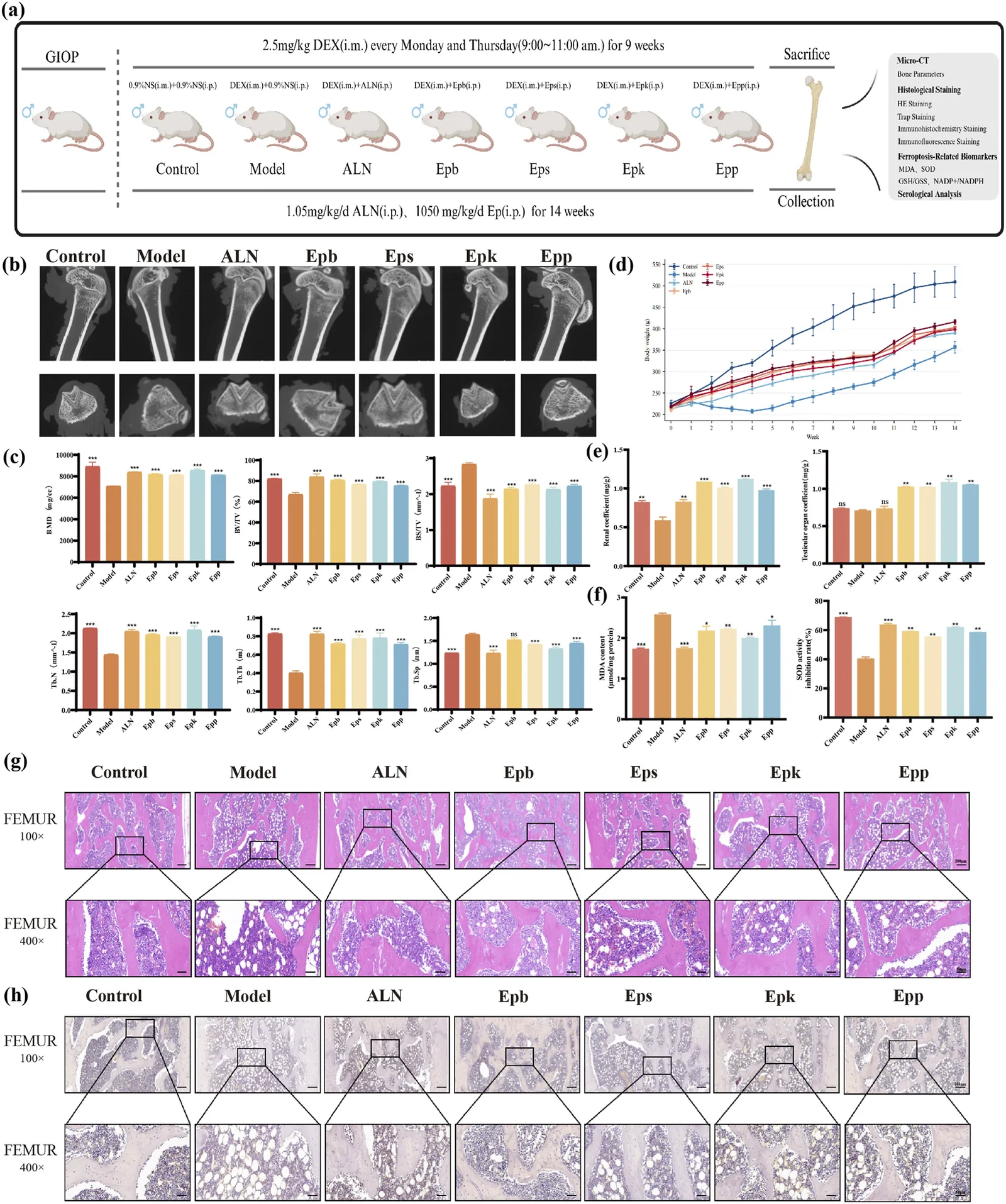
Epimedium treatment delays bone loss induced by GIOP in rats. **(a)** Schematic diagram of the *in vivo* experiments. Created in BioRender. LI, P. (2026). https://BioRender.com/q7dqv7o. **(b)** Representative micro-CT images showing that femoral bone loss was prevented by Epimedium administration. **(c)** Quantitative analyses of bone microstructural parameters, including BMD, BV/TV, BS/TV, Tb.N, Tb.Th, and Tb.Sp. **(d)** Body weight of the rats in each group. **(e)** Organ coefficients of kidneys and testes in each group of rats. **(f)** MDA and SOD levels in rat femurs across groups. **(g)** H&E staining of femoral tissues in different groups after Epimedium intervention. **(h)** TRAP staining of femoral tissues in different groups after Epimedium intervention. All bar graphs are presented as the mean ± SD. *p < 0.05, **p < 0.01, ***p < 0.001 compared with the Model group, n = 10 per group. BMD, bone mineral density; BV/TV, bone volume per tissue volume; BS/TV, bone surface area to tissue volume ratio; Tb.N, trabecular number; Tb.Th, trabecular thickness; Tb. Sp, trabecular separation.

Micro-CT analysis revealed impaired femoral microarchitecture in GIOP rats, which was restored by Epimedium treatment (Figure 1b). Quantitative assessments (Figure 1c) indicated elevatedbone mineral density (BMD), bone volume fraction (BV/TV), trabecular number (Tb.N), and trabecular thickness (Tb.Th), concomitant with reduced trabecular separation (Tb.Sp) relative to the Model group following Epimedium administration. These findings point to reversal of bone loss and enhancement of bone connectivity by Epimedium.

Histopathological evaluation of H&E staining (Figure 1g)revealed sparse, fractured trabeculae alongside diminished osteocyte density in the Model group. By contrast,all Epimedium-treated groups, most notably Epb and Epk, exhibitedelevated trabecular number, improved continuity, and strengthened bone mineralization. TRAP staining (Figure 1h)substantiated heightened osteoclast (TRAP + cells) activity in the Model group, which was markedly suppressed upon Epimedium treatment, implying inhibition of osteoclast-mediated bone resorption.

Moreover, Epimedium administration reduced oxidative stress in bone tissue, as reflected by diminished MDA content and elevated SOD activity relative to the Model group (Figure 1f).

### 3.2 Epimedium treatment delays GIOP in rats in terms of bone formation and bone resorption

To assess the effects of Epimedium on bone metabolism, key biomarkers of bone formation and resorption were analyzed. IHC findings indicated that administration of various Epimedium extracts upregulated the osteogenic proteins osteocalcin (OCN), osteoprotegerin (OPG), and Runt-related transcription factor 2 (RUNX2) in the femurs of GIOP rats relative to the DEX-only Model group (Figures 2a–c). Aligning with IHC observations, serum OPG and alkaline phosphatase (ALP) levels, which were substantially reduced in the Model group, were restored following Epimedium administration, pointing to strengthened osteoblast activity and bone formation capacity (Figures 2d,e). Epk demonstrated the most prominent effect in raising OPG and ALP levels under the present equal-mass dosing regimen.

**FIGURE 2:**
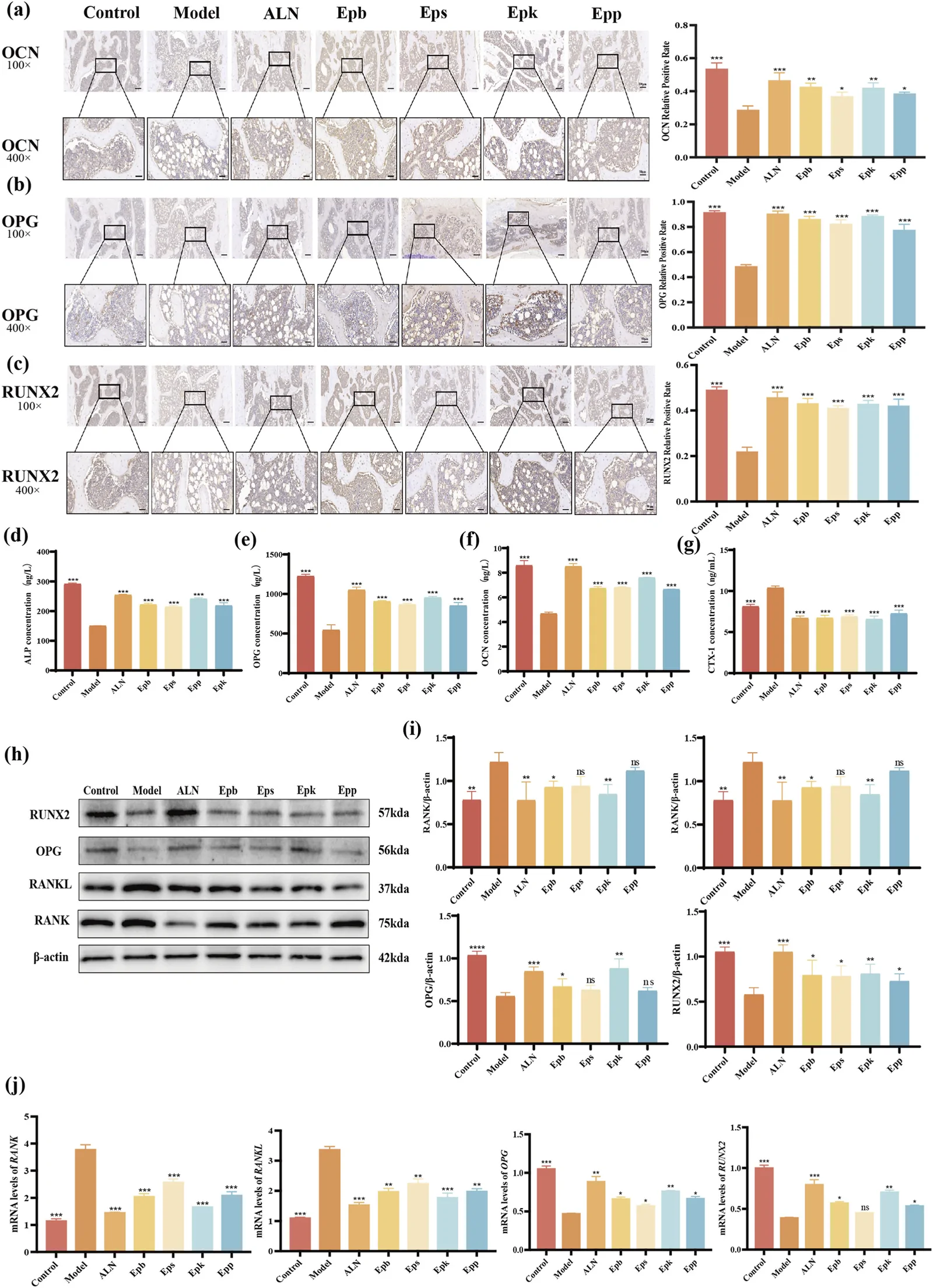
Epimedium treatment delays GIOP in rats in terms of bone formation and bone resorption. **(a)** IHC staining for OCN of decalcified bone sections. **(b)** IHC staining for OPG of decalcified bone sections. **(c)** IHC staining for RUNX2 of decalcified bone sections. **(d)** Detection of serum content of ALP. **(e)** Detection of serum content of OPG. **(f)** Detection of serum content of OCN. **(g)** Detection of serum content of CTX-1. **(h–j)** Detection of RANK, RANKL, OPG, and RUNX2 protein and mRNA expression in rat femurs using Western blotting and qPCR. All bar graphs are presented as the mean ± SD. *p < 0.05, **p < 0.01, ***p < 0.001 compared with the Model group, n = 6 per group.

Concurrently, Epimedium treatment suppressed bone resorption, as evidenced by a marked reduction in serum C-terminal telopeptide of type I collagen (CTX-I) levels relative to the Model group (Figure 2g). Western blot and qPCR analyses additionally showed that Epimedium extracts elevated protein and mRNA expression of OPG and RUNX2while lowering the expression of receptor activatorof nuclear factor-κB (RANK) together with its ligand RANKL (Figures 2h–j), pointing to a shift in the RANK/RANKL/OPG pathway toward suppression of osteoclast differentiation and activity.

In the experiments described above, we evaluated the therapeutic effects of Epimedium on GIOP by monitoring the general condition of the rats in each group, recording body weight and organ coefficients, and examining H&E and TRAP staining of femoral sections. During treatment, Epimedium improved the clinical manifestations of GIOP, increased body weight and kidney coefficient, regulated the bone formation and resorption markers (ALP, OPG, OCN, and CTX-1), and upregulated the osteogenesis-related protein RUNX2. Through the RANK/RANKL/OPG and RUNX2 pathways, Epimedium promoted osteogenic proteins in bone tissue while inhibiting osteoclast-related proteins, thereby ameliorating osteoporosis by promoting bone formation and suppressing bone resorption. Among the four Epimedium groups, Epk showed the most pronounced bone-regulating effect under the present equal-dose regimen, exhibiting the strongest anti-GIOP activity in this experimental setting.

Taken together, these findings demonstrate that Epimedium counteracts GIOP progression by simultaneously stimulating osteoblast-driven bone formation and suppressing osteoclast-mediated bone resorption, with Epk showing the most pronounced overall effects among the four species examined under the present equal-mass dosing regimen.

### 3.3 Proteomic analysis reveals functional alterations in GIOP rats and response to epk treatment

Principal component analysis (PCA) yielded clear separation among Control, Model, ALN, and Epk groups, pointing to distinct protein expression profiles across treatment conditions (Supplementary Figure S3). Volcano plot analysis identified 876 differentially expressed proteins (DEPs) between the Model and Control groups (defined as |fold change| ≥ 1.2 with P < 0.05; peptide and protein FDR <1%), comprising 458 downregulated and 418 upregulated entities (Supplementary Figure S4). Hierarchical clustering analysis of the DEPs is shown in Figure 3a. ALN and Epk interventions altered protein expression, reversing 345 and 368 DEPs, respectively, relative to the Model group. Here, “reversed” denotes DEPs that were significantly altered in the Model versus Control comparison and whose expression was significantly restored toward the Control level in the Epk (or ALN) versus Model comparison (opposite direction of change, |fold change| ≥ 1.2, P < 0.05).

**FIGURE 3:**
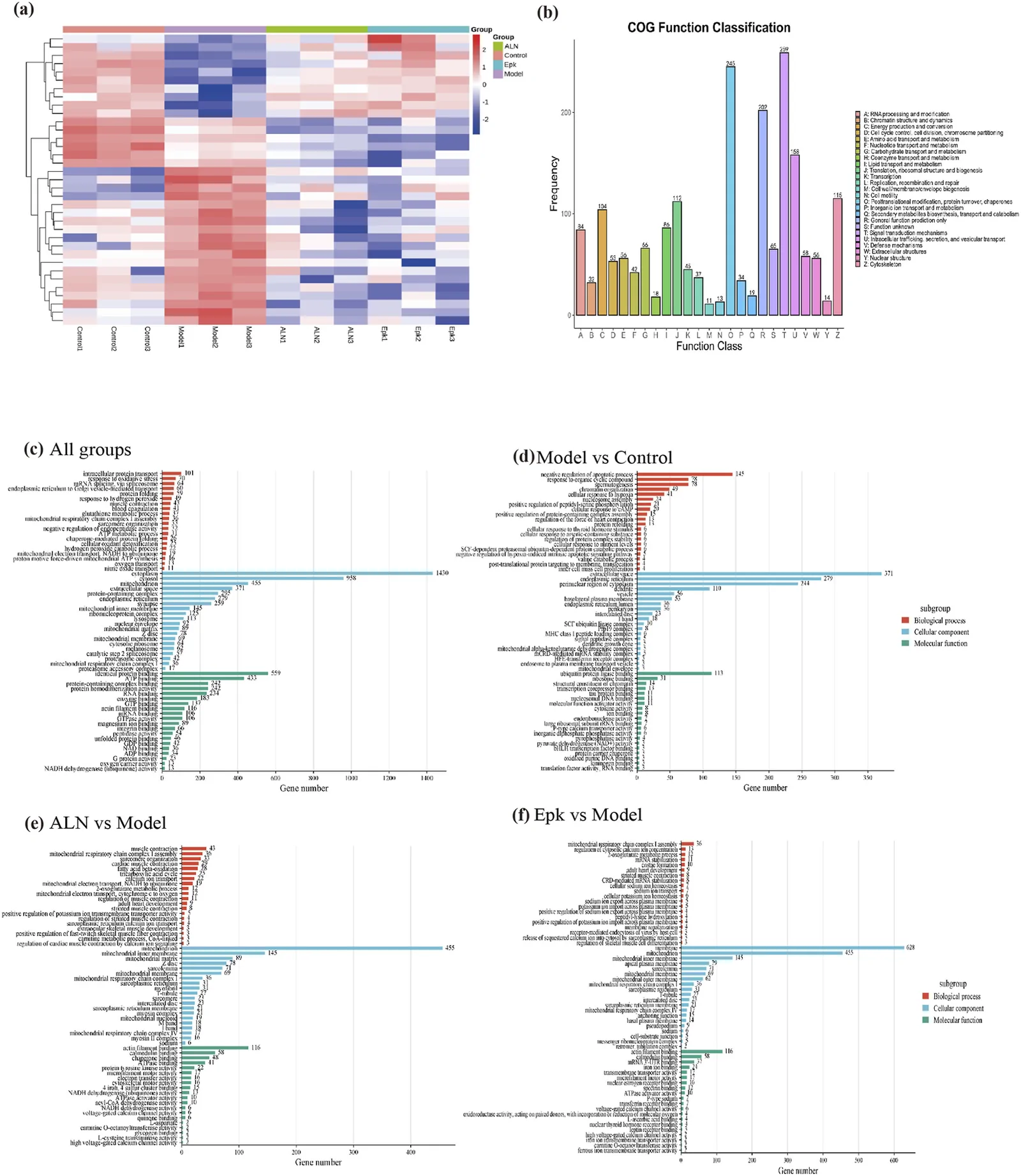
Proteomic analysis reveals functional alterations in GIOP rats and response to Epk treatment. **(a)** Hierarchical clustering analysis of all samples. **(b)** COG classification analysis of DEPs. **(c)** GO functional annotation analysis of all DEPs. **(d)** GO functional annotation analysis of DEPs in the Model group versus the Control group. **(e)** GO functional annotation analysis of DEPs in the ALN treatment group versus the Model group. **(f)** GO functional annotation analysis of DEPs in the Epk treatment group versus the Model group.

COG functional classification of DEPs revealed their predominant involvement inamino acid transport and metabolism, lipid transport and metabolism, alongside signal transduction (Figure 3b). GO enrichment analysis of DEPs spanning all comparisons uncovered substantial terms associated with GIOP pathophysiology and treatment response. In the Model versus Control comparison, key enriched terms encompassed negative regulation of apoptosis (biological process (BP)), extracellular matrix (cellular component (CC)), and ubiquitin-protein ligase binding (molecular function (MF)) (Figure 3d). Subsequent to Epk treatment,the most significantly enriched GO terms shifted toward processes and components encompassingmitochondrial respiratory chain complex I (BP), membrane (CC), and actin filament binding (MF) (Figure 3f), implying a therapeutic effect mediated through energy metabolism and cytoskeletal regulation.

The comprehensive GO analysis encompassing all DEPs further emphasized the significance of protein transport, oxidative stress response (BP), cytoplasm, and mitochondria (CC) within the GIOP process.

### 3.4 KEGG analysis reveals synergistic interactions of oxidative phosphorylation and ferroptosis pathways in GIOP

KEGG pathway enrichment analysis revealed that, relative to the control group,the DEPs in the GIOP Model group were significantly enriched in oxidative phosphorylation and ferroptosis signaling pathways. In the ALN versus Model comparison, DEPs were mainly enriched inoxidative phosphorylation and fatty acid metabolism pathways. In contrast,Epk treatment considerably reversed the protein expression abnormalities in the oxidative phosphorylation and ferroptosis pathways observed in the Model group (Figure 4). Comprehensive enrichment analysis of all differentially expressed proteins further validated that oxidative phosphorylation, glutathione metabolism, ferroptosis signaling, and fatty acid metabolism constitute a core synergistic regulatory network within the pathological process of GIOP.

**FIGURE 4:**
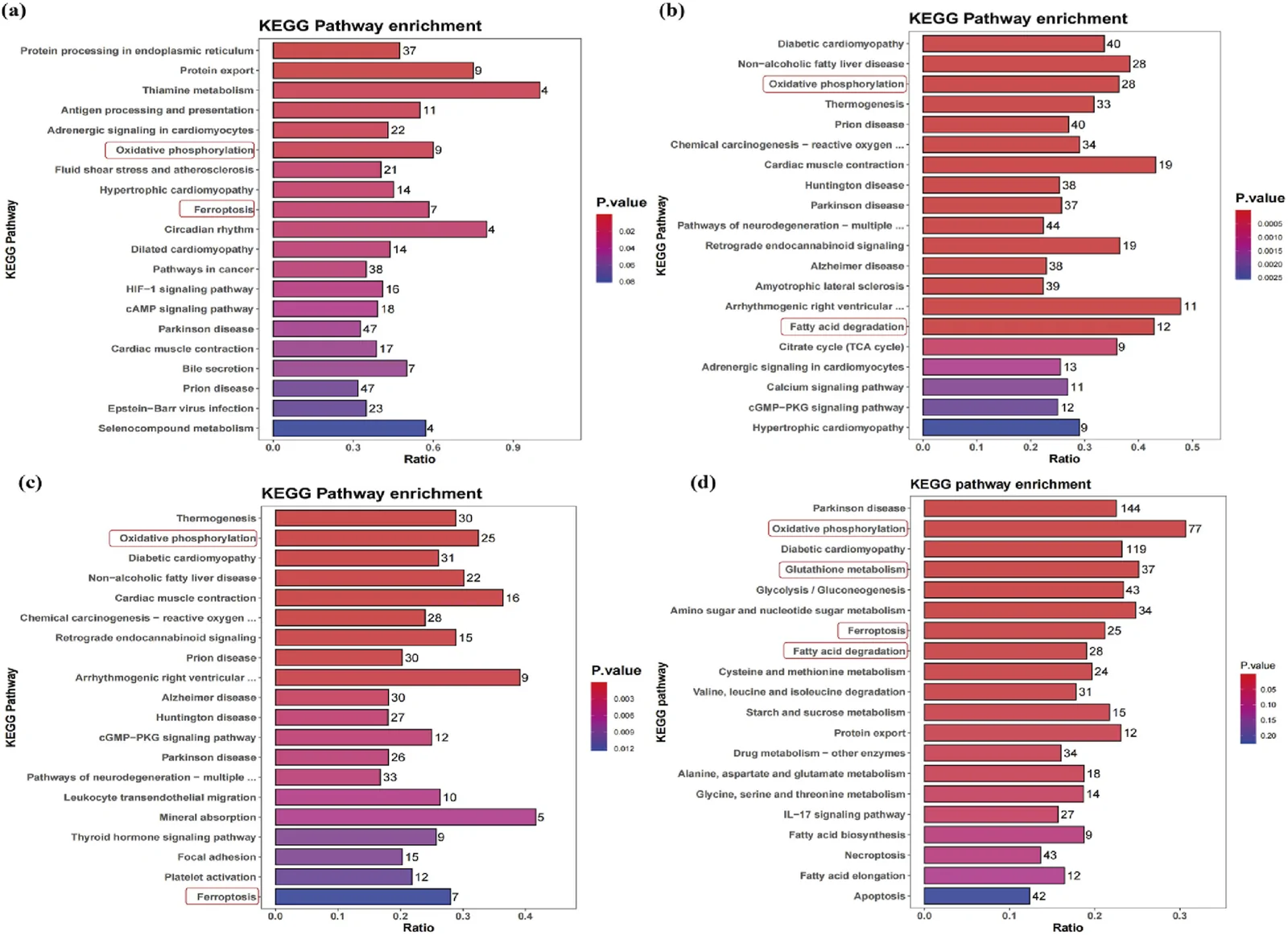
Oxidative phosphorylation, glutathione metabolism, ferroptosis signaling pathways, and fatty acid metabolism pathways participate in the pathological process of GIOP through synergistic interactions. **(a)** KEGG pathway enrichment analysis of DEPs in the Model group versus the Control group. **(b)** KEGG pathway enrichment analysis of DEPs in the ALN treatment group versus the Model group. **(c)** KEGG pathway enrichment analysis of DEPs in the Epk treatment group versus the Model group. **(d)** KEGG pathway enrichment analysis of all DEPs identified across the comparative groups.

These pathways engage in interaction through the following mechanisms: Oxidative phosphorylation, serving as the core of cellular energy generation, provides the ATP and reducing equivalents required for cellular defense responses, while also acting asa substantial source of reactive oxygen species (ROS). Fatty acid metabolism suppliesthe key substrates for ferroptosis, namely, polyunsaturated fatty acids (PUFAs). These PUFAs undergo esterification into membrane phospholipids under catalysis by enzymes such as ACSL4 and LPCAT3, rendering them vulnerable to lipid peroxidation attacks. Glutathione metabolism directly scavenges peroxides through maintenance of GPX4 activity, thereby suppressing ferroptosis. Upon GPX4 inactivation or glutathione depletion, ROS generated from oxidative phosphorylation, combined with PUFAs supplied by fatty acid metabolism, trigger a chain reaction of lipid peroxidation in the presence of iron ions, ultimately culminating in cellular ferroptosis.

These pathways collectively constitute a dynamic interactive network: Fatty acid beta-oxidation supplies substrates for oxidative phosphorylation and enhances electron transport chain activity, while oxidative phosphorylation, in turn, provides supportfor fatty acid metabolism and glutathione synthesis. The imbalance of this network plays a critical role in GIOP-induced bone loss. Epk treatment can restore metabolic homeostasis and inhibit ferroptosis by simultaneously modulating these multiple pathways.

### 3.5 Epk alleviates GIOP through regulation of lipid and amino acid metabolism pathways

Untargeted metabolomics analysis revealed clear separation of metabolic profiles among the Control, Model, ALN, and Epk groups by PCA (Figures 5a,c), indicating significant alterations in the metabolic state of bone tissue in GIOP rats. OPLS-DA further validated intergroup differences with good model stability, and model robustness was confirmed by 200× permutation testing, with the OPLS-DA score plots shown in Supplementary Figure S5 and the R^2^ and Q^2^ values reported in Supplementary Table S3. For all six models, the permutation p values for Q^2^ were below 0.05, whereas those for R^2^Y were above 0.05; this pattern is common in small-sample OPLS-DA analyses and indicates reliable predictive ability of the models, although the explained variance is subject to stochastic fluctuation. Comparative analysis of metabolite expression trends identified 153 common differential metabolites. Among these, ALN and Epk interventions reversed 137 and 127 abnormally expressed metabolites in the Model group, respectively (defined as metabolites significantly altered in the Model versus Control comparison that were significantly restored toward the Control level after treatment, VIP >1, |fold change| ≥ 1.2, P < 0.05; Supplementary Table S4).

**FIGURE 5:**
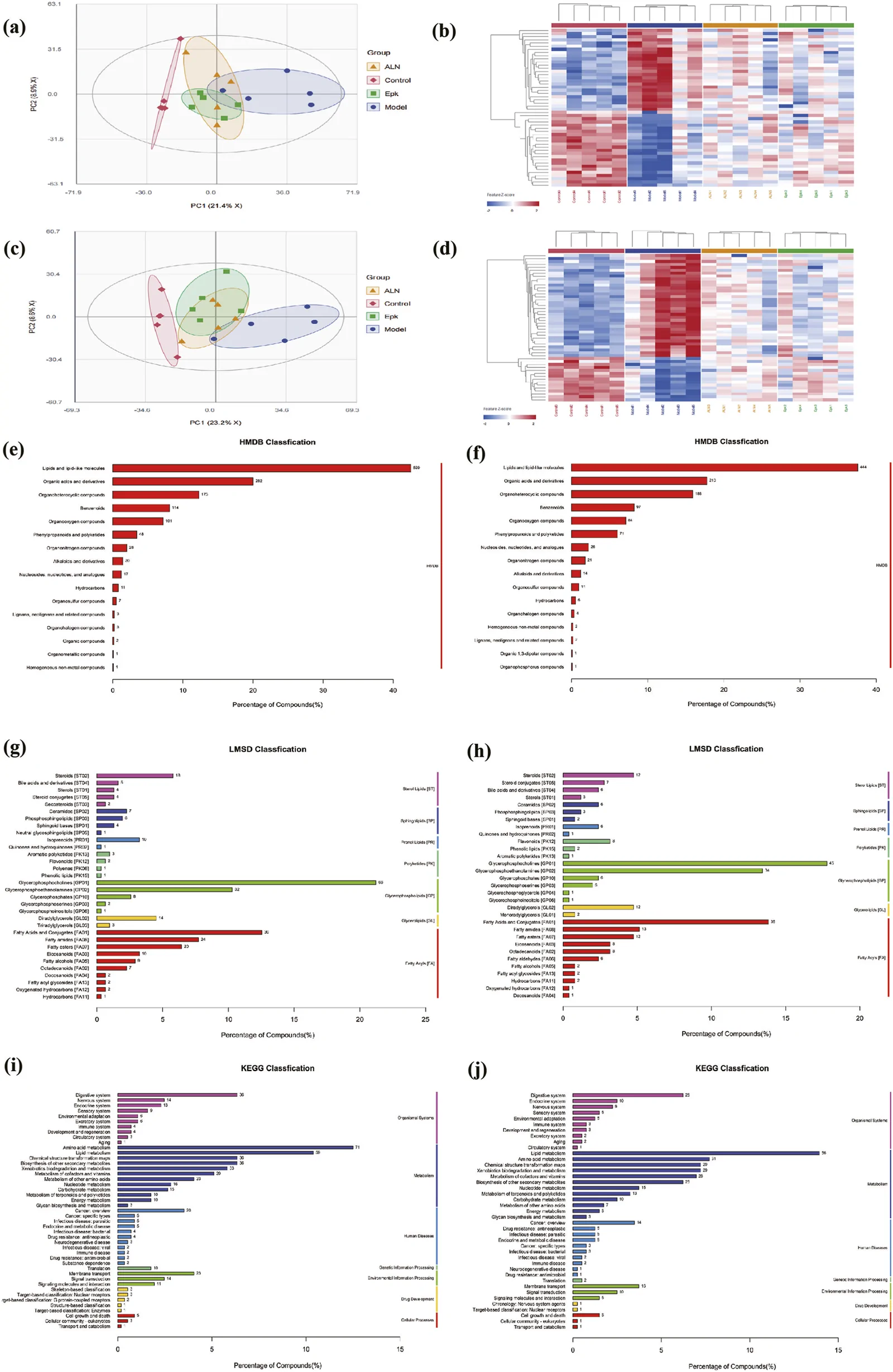
Epk primarily exerts its GIOP-delaying effects through lipid metabolism and amino acid metabolism pathways. **(a)** PCA score plot of samples analyzed in positive ion mode (ESI+). **(b)** Hierarchical clustering analysis of all samples analyzed in positive ion mode (ESI+). **(c)** PCA score plot of samples analyzed in ESI-. **(d)** Hierarchical clustering analysis of all samples analyzed in ESI-. **(e)** Identification of differential metabolites in ESI + using the HMDB. **(f)** Identification of differential metabolites in ESI- using the HMDB. **(g)** Classification of differential metabolites in ESI + using the LMSD. **(h)** Classification of differential metabolites in ESI- using the LMSD. **(i)** KEGG pathway enrichment analysis of all differential metabolites identified in ESI+. **(j)** KEGG pathway enrichment analysis of all differential metabolites identified in ESI-.

HMDB annotation showed that these differential metabolites were primarily classified as lipids and lipid-related molecules, organic acids and their derivatives (Figures 5e,f). Further analysis using the LMSD lipid database revealed that the differential metabolites encompassed several subclasses including sterol lipids, sphingolipids, glycerophospholipids, and fatty acids (Figures 5g,h). KEGG pathway enrichment analysis suggested that Epk exerts its therapeutic effects mainly by regulating lipid metabolism and amino acid metabolism pathways, while also involving nucleotide metabolism and membrane transport processes (Figures 5i,j).

These results demonstrate a severe disruption of metabolic homeostasis in bone tissue during GIOP progression. Epk treatment reversed GIOP-induced metabolic disturbances through multi-target regulationof the lipid peroxidation-related metabolic network.

### 3.6 Integrated analysis of proteomics and metabolomics

Spearman’s rank correlation analysis was performed on differentially expressed proteins and metabolites from proteomics and metabolomics data, with a heatmap of correlation coefficients generated. As shown in Figure 6, the analysis indicated significant correlations between differentially expressed proteins and metabolites. Significantly correlated differential metabolites and proteins with absolute Spearman correlation coefficients greater than 0.5 were screened. The differential metabolites were primarily concentrated in lipid metabolism and amino acid metabolism pathways, while the differential proteins were mainly implicated in oxidative phosphorylation, glutathione metabolism, ferroptosis signaling pathways, and fatty acid metabolism signaling pathways. Among these, the expression changes of GSS, GPX4, ACSL4, HO-1 (HMOX1), FTH1, LPCAT3, and STEAP3, and other ferroptosis-related molecules, reflected the coordinated regulation of ferroptosis pathways in Epk-treated GIOP rats. These molecules serve as important targets for studying ferroptosis mechanisms and developing ferroptosis-related therapeutic strategies to intervene in the ferroptosis process in GIOP rats.

**FIGURE 6:**
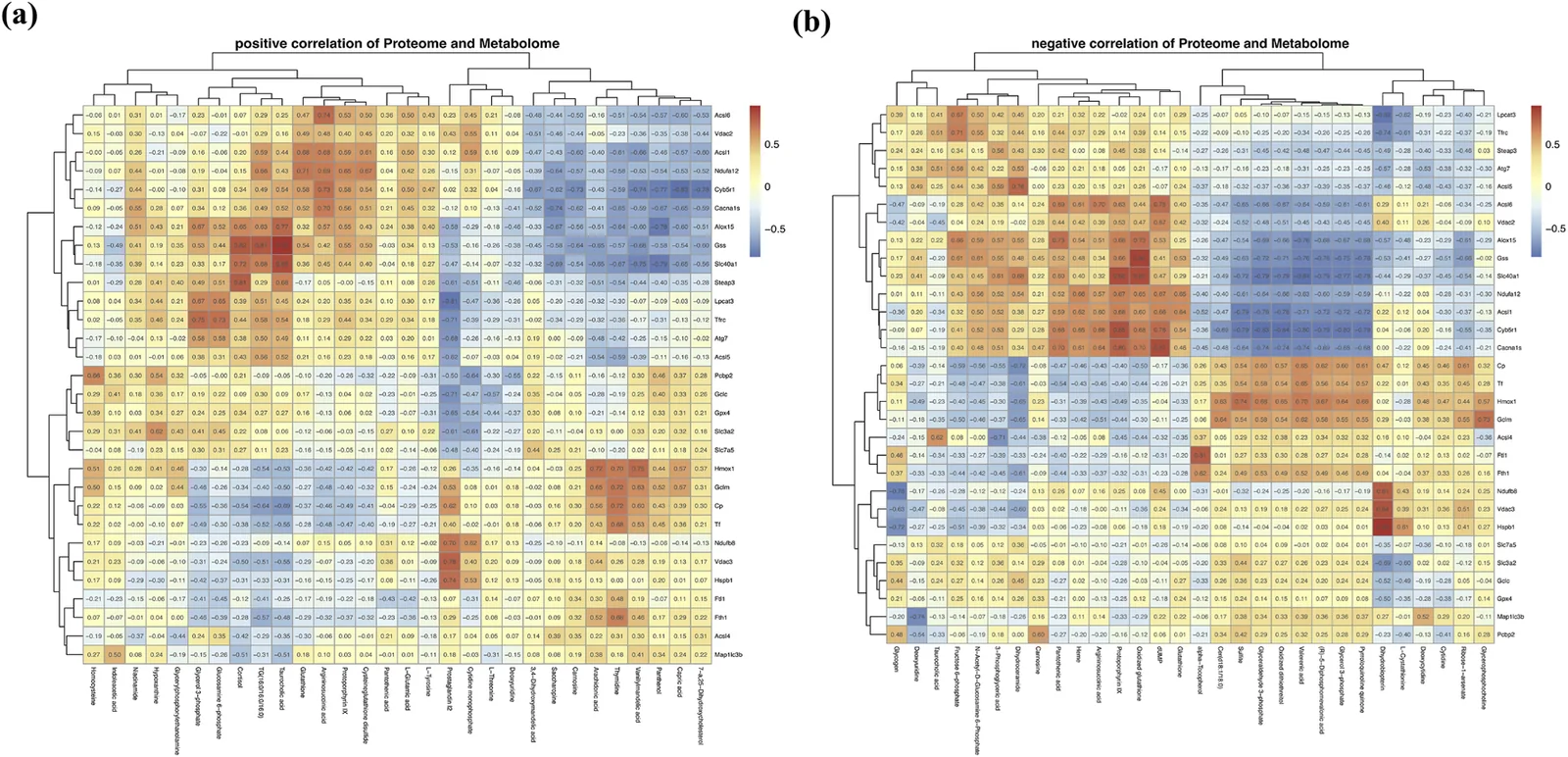
Integrated analysis of proteomics and metabolomics. **(a)** Correlation analysis between differentially expressed proteins and differential metabolites identified in ESI+. **(b)** Correlation analysis between differentially expressed proteins and differential metabolites identified in ESI-.

### 3.7 Epk alleviates GIOP by activating the KEAP1/NRF2 pathway and inhibiting ferroptosis

Immunohistochemical analysis revealed that the GIOP Model group exhibited significantly decreased levels of NRF2 and its downstream effector HO-1, along with increased KEAP1 expression in femoral tissue compared to the control group (Figures 7a–c). Epk treatment effectively reversed these alterations, consistent with activation of the KEAP1/NRF2/HO-1 antioxidant pathway.

**FIGURE 7:**
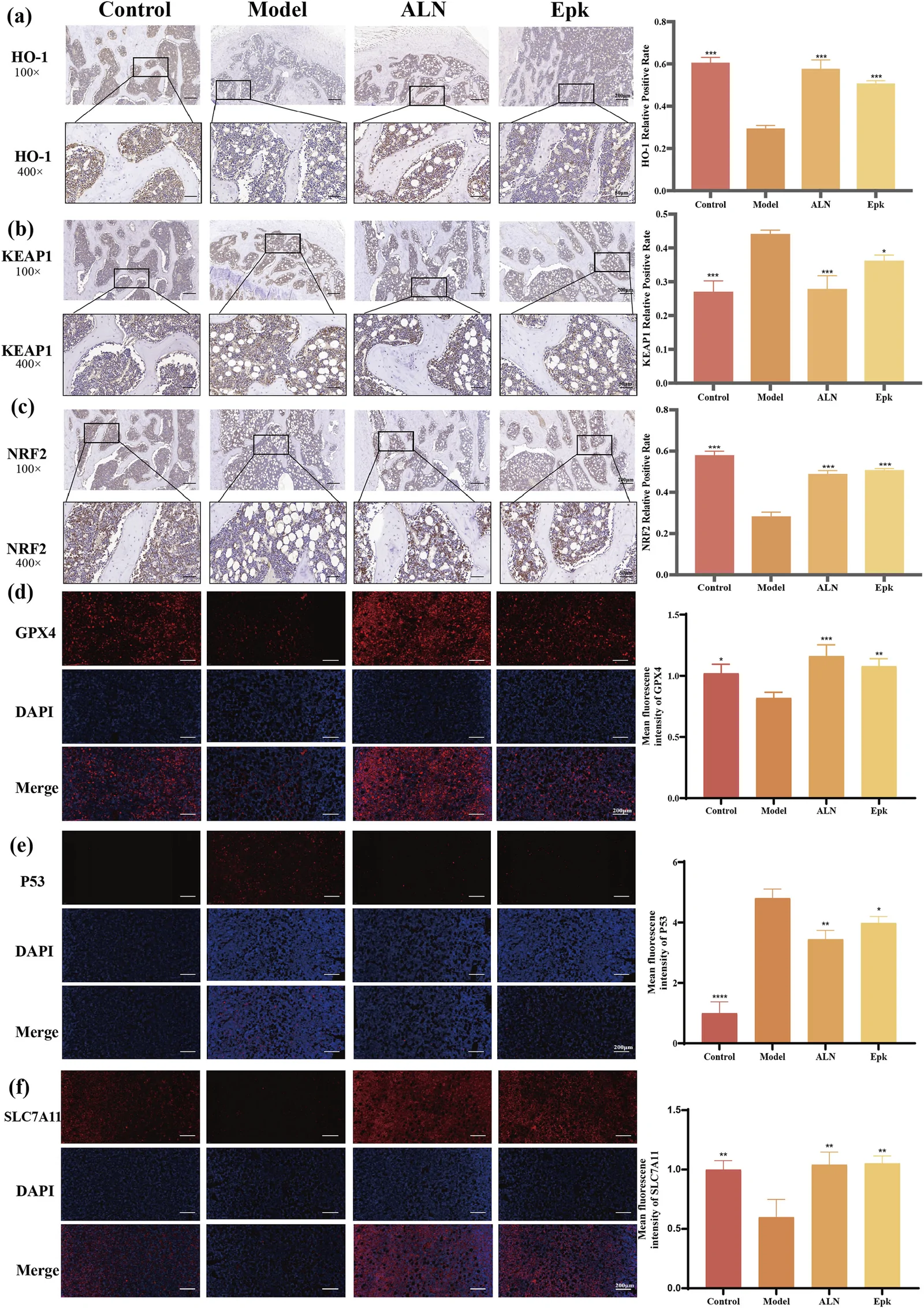
Epk improves GIOP in rats by regulating oxidative stress levels. **(a)** IHC staining for HO-1 of decalcified bone sections. **(b)** IHC staining for KEAP1 of decalcified bone sections. **(c)** IHC staining for NRF2 of decalcified bone sections. **(d)** IF staining for GPX4 of decalcified bone sections. **(e)** IF staining for P53 of decalcified bone sections. **(f)** IF staining for SLC7A11 of decalcified bone sections. All bar graphs are presented as the mean ± SD. *p < 0.05, **p < 0.01, ***p < 0.001 compared with the Model group, n = 6 per group.

Immunofluorescence detection further demonstrated that Epk administration significantly upregulated the expression of ferroptosis biomarkers GPX4 and SLC7A11 in bone tissue, while suppressing P53 levels (Figures 7d–f). GPX4 was distributed in both cortical and trabecular bone regions. Compared to the model group, both ALN and Epk treatments markedly reduced ferroptosis levels, with Epk showing comparable efficacy to ALN.

These findings are collectively consistent with Epk ameliorating GIOP by enhancing cellular antioxidant capacity through KEAP1/NRF2/HO-1 pathway activation and concurrently suppressing ferroptosis via the SLC7A11/GPX4 axis, although causal confirmation requires targeted loss-of-function and rescue experiments.

### 3.8 Epk alleviates GIOP through the KEAP1/NRF2/SLC7A11/GPX4 signaling axis

Biochemical analysis showed that Epk treatment modulated key ferroptosis-related markers in GIOP rats. Although serum GSH levels were elevated in the Model group relative to controls, Epk intervention effectively reduced this elevation (Figure 8a). The apparent inconsistency between elevated serum GSH and the reduced tissue-level GSH/GSSG ratio in the model group likely reflects the fact that circulating GSH mainly mirrors systemic (largely hepatic) thiol status, whereas the tissue redox imbalance is captured by the femoral GSH/GSSG and NADP+/NADPH ratios; this interpretation is elaborated in the Discussion. Epk administration also elevated serum GPX4 levels (Figure 8b), which were suppressed in Model rats. The GSH/GSSG and NADP+/NADPH ratios, serving as critical indicators of cellular redox balance, were diminished in the Model group but substantially restored by Epk treatment (Figures 8c,d). In addition, femoral 4-HNE content and Fe^2+^ levels, direct indicators of lipid peroxidation and iron accumulation, were markedly elevated in the Model group relative to the Control group and were significantly reduced following Epk treatment (Figures 8e,f).

**FIGURE 8:**
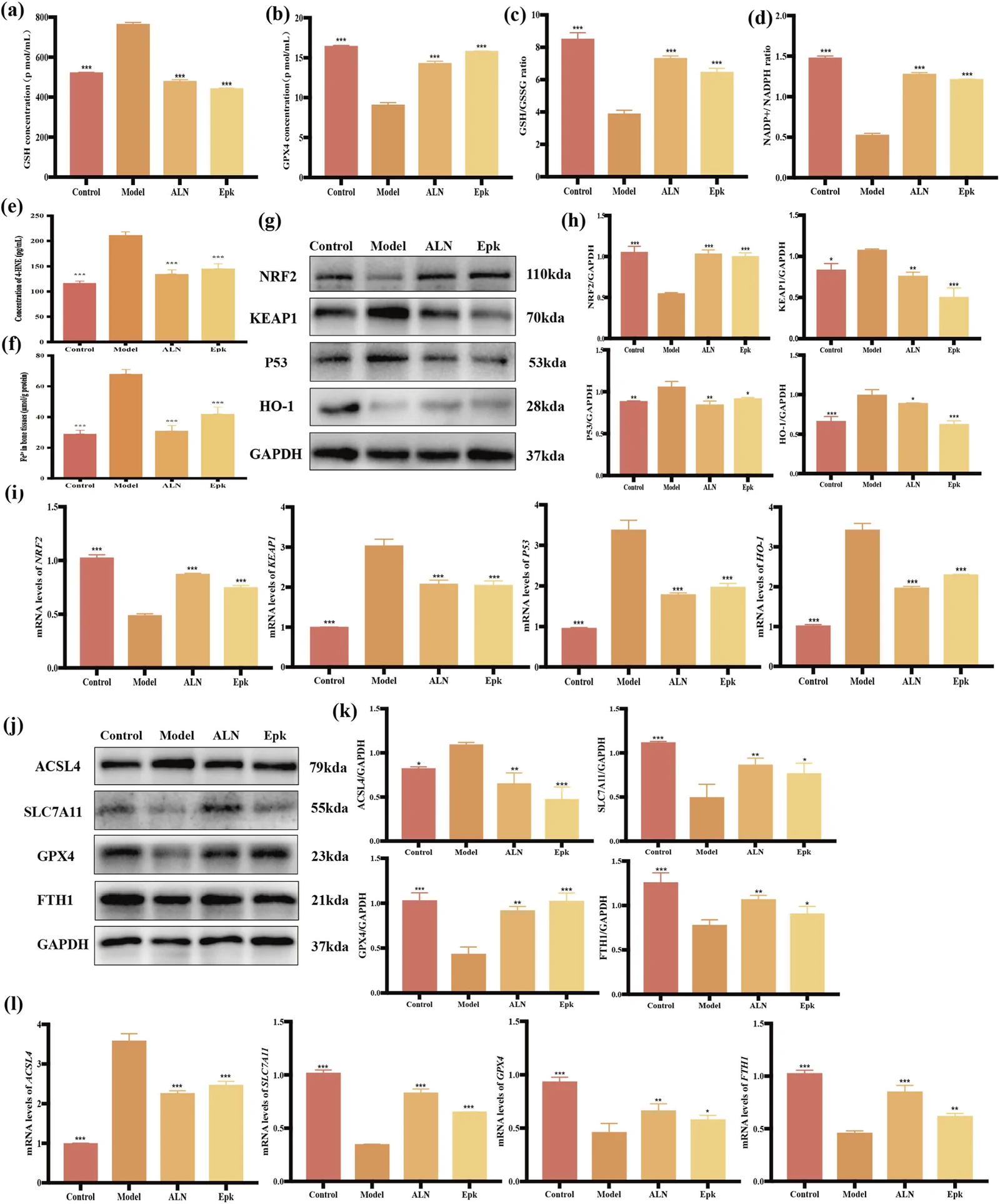
Epk delays GIOP in rats via the KEAP1/NRF2/SLC7A11/GPX4 signaling pathway. **(a)** Detection of serum content of GSH. **(b)** Detection of serum content of GPX4. **(c)** Results of GSH/GSSG levels in rat femurs across groups. **(d)** Results of NADP+/NADPH levels in rat femurs across groups. **(e)** Results of 4-HNE levels in rat femurs across groups. **(f)** Results of Fe^2+^ levels in rat femurs across groups. **(g–l)** Detection of NRF2,KEAP1,P53,HO-1,ACSL4,SLC7A11,GPX4 and FTH1 Protein and mRNA Expression in rat femurs Using Western blotting and qPCR. All bar graphs are presented as the mean ± SD. *p < 0.05, **p < 0.01, ***p < 0.001 compared with the Model group, n = 6 per group.

Western blot and qPCR analyses uniformly showed that Epk treatment elevated protein and mRNA expression of GPX4, NRF2, SLC7A11, and HO-1 in femoral tissue, while lowering ACSL4, FTH1, KEAP1, and P53 levels relative to the model group (Figure 8g–l).

Taken together, these data support, though they do not definitively establish, that Epk exerts its anti-osteoporotic effects through modulation of the KEAP1/NRF2/SLC7A11/GPX4 signaling pathway, thereby reinstating redox homeostasis and suppressing ferroptosis-related signaling in bone tissue.

## 4 Discussion

GIOP constitutes a substantial clinical complication linked to prolonged GCs therapy, manifesting through an imbalance in bone remodeling thatchiefly favors bone resorption over formation (Humphrey et al., 2023; Shi et al., 2025; Tong et al., 2025). While the skeletal consequences of glucocorticoid excessare thoroughly documented, molecular mechanisms underlying GIOP pathogenesis remain incompletely elucidated, particularly with respect to the involvement of regulated cell death pathways in bone deterioration. This comprehensive investigation furnishes compelling evidence that Epimedium koreanum Nakai (Epk), a traditional medicinal herb, confers substantial protection against GIOP through multifaceted mechanisms centered uponsuppression of ferroptosis, an iron-dependent form of regulated cell death driven by lipid peroxidation.

Regarding the novelty of the present study, although a previous report demonstrated that mangiferin attenuates osteoporosis by inhibiting osteoblastic ferroptosis through the KEAP1/NRF2/SLC7A11/GPX4 pathway (Deng et al., 2024), the present study differs in several important respects. First, we comparatively evaluated the anti-osteoporotic effects of the four Epimedium species listed in the Chinese Pharmacopoeia (2025 edition) under the same experimental conditions, and Epk showed the most pronounced effects in this experimental setting. Second, rather than focusing on a single molecular axis, we performed 4D label-free proteomics and untargeted metabolomics on femoral tissue from the Control, Model, ALN, and Epk groups, delineating a synergistic network of oxidative phosphorylation, glutathione metabolism, fatty acid metabolism, and ferroptosis in GIOP, and we validated the key hub molecules *in vivo*. Third, we studied the clinically relevant glucocorticoid (dexamethasone)-induced osteoporosis model, in which ferroptosis inhibition is emerging as a key mechanism. Fourth, in contrast to the osteoblast-specific claim of the earlier report, our conclusions are explicitly framed at the tissue level, acknowledging the whole-bone composition of the assays. Collectively, these features distinguish the present work while corroborating the emerging role of ferroptosis in osteoporosis.

Our study demonstrates that Epk treatment effectively preserves bone microarchitecture in a rat model of GIOP, as evidenced by the maintenance of bone mineral density, trabecular structure parameters, and bone volume fraction. These structural improvements correlate with a rebalancing of bone turnover markers, where Epk promotes osteoblastic activity through the upregulation of key osteogenic factors including OCN, OPG, and transcription factor RUNX2, while simultaneously suppressing osteoclastic bone resorption as indicated by reduced CTX-I levels and modulation of the RANK/RANKL signaling pathway. Epk exhibited the most pronounced therapeutic efficacy among the four Epimedium variants examined under the present equal-mass dosing regimen, which may partly reflect differences in origin-specific phytochemical composition. However, because direct pairwise statistical comparisons among the Epimedium groups and comparative phytochemical standardization were not performed, the intrinsic superiority of Epk cannot be conclusively established; this highlights the importance of chemical standardization in herbal medicine applications.

The integration of proteomic and metabolomic analyses in this study reveals ferroptosis as a central pathological process in GIOP development. The significant enrichment of differentially expressed proteins and metabolites inferroptosis-associated pathways, including oxidative phosphorylation, glutathione metabolism, and fatty acid metabolism, indicatesa complex metabolic adaptation to glucocorticoid exposure. Specifically, we observed substantial dysregulation of key ferroptosis regulators, with compromised GPX4 function and disrupted glutathione homeostasis creating conditions favorable for lipid peroxide accumulation and subsequent ferroptotic cell death in bone tissue. This metabolic dysregulation extends to altered amino acid and lipid profiles, where Epk treatment effectively normalizes the accumulation of peroxidation-susceptible polyunsaturated fatty acids while restoring redox balance throughcorrection of NADP+/NADPH and GSH/GSSG ratios.

We also note that serum GSH was elevated in the Model group relative to Control and was reduced by Epk treatment (Figure 8a). At first glance this appears inconsistent with a ferroptosis-permissive, glutathione-depleted state. However, circulating GSH primarily reflects systemic (largely hepatic) thiol mobilization and export rather than the redox status of bone tissue itself. In the present study, tissue-level indices (femoral GSH/GSSG and NADP+/NADPH ratios) were reduced in the Model group and restored by Epk, and femoral GPX4 was increased by Epk, indicating a bone-tissue-specific redox imbalance that is masked by systemic GSH levels. Elevated serum GSH in the Model group may therefore represent a compensatory systemic antioxidant response to glucocorticoid-induced oxidative stress, and its normalization by Epk may reflect attenuation of this systemic stress. Direct measurement of tissue glutathione and thiol pools will be necessary to confirm this interpretation.

At the molecular level, Epk treatment was associated with changes consistent with a dual mechanism of action, namely, concurrent activation of the KEAP1/NRF2 antioxidant pathway and enhancement of the SLC7A11/GPX4 anti-ferroptotic axis, although causality cannot be established from the present data. The coordinated upregulation of NRF2 and its downstream targets strengthens cellular defenses against oxidative stress, while the induction of SLC7A11 expression supports cystine uptake and glutathione biosynthesis, thereby providing essential substrates for GPX4-mediated reduction of phospholipid hydroperoxides. This comprehensive modulation is consistent with interruption of the propagation of lipid peroxidation that would otherwise culminate in ferroptosis. Additional changes in key ferroptosis modulators, including reduced ACSL4 and P53 alongside reduced FTH1, further support the proposed anti-ferroptotic effect of Epk. Of note, the interpretation of the FTH1 change remains cautious: FTH1 is primarily involved in iron storage, and its role in ferroptosis is context-dependent, so direct iron-related measurements are required to substantiate this interpretation.

The implications of these findings extend beyond the specific context of GIOP management. The identification of ferroptosis as a targetable mechanism in glucocorticoid-induced bone loss opens new therapeutic avenues for osteoporosis treatment more broadly. While conventional therapies primarily focus on inhibiting bone resorption, the multifaceted approach of Epk, simultaneously enhancing bone formation, suppressing oxidative stress, and preventing ferroptosis, may offer superior clinical outcomes, particularly in advanced cases where multiple pathological processes converge. The metabolic reprogramming induced by Epk treatment suggests that cellular bioenergetics and redox homeostasis play previously underappreciated roles in bone integrity maintenance under glucocorticoid stress conditions. In addition, P53 was assessed as one of several ferroptosis-associated markers; because P53 can exert context-dependent and dual roles in ferroptosis, our findings regarding P53 are interpreted conservatively as supporting evidence rather than as a specific indicator of ferroptosis.

Our multi-omics approach further reveals that Epk’s therapeutic effects involve the regulation of critical biological processes including protein transport, oxidative stress response, and cellular component organization, with significant enrichment in cytoplasmic and mitochondrial functions. The observed alterations in oxidative phosphorylation and glutathione metabolism pathways provide mechanistic insights into how Epk maintains energy homeostasis and redox balance in bone tissue. The correlation analysis between proteomic and metabolomic data confirms the coordinated regulation of key ferroptosis biomarkers, including GSS, GPX4, ACSL4, HO-1, FTH1, LPCAT3, and STEAP3, in Epk-treated GIOP rats.

The observed changes in the KEAP1-NRF2/HO-1 signaling pathway represent another important aspect of the proposed mechanism. This pathway serves as a vital endogenous antioxidant system; in the present study, Epk treatment was associated with enhanced expression of NRF2 and its downstream targets, which is consistent with mitigation of ROS accumulation and cellular damage, although direct proof of functional NRF2 activation (e.g., nuclear translocation) is not provided. The simultaneous regulation of SLC7A11, a core component of the cystine/glutamate reverse transporter, further supports Epk’s comprehensive approach to maintaining cellular redox homeostasis and preventing ferroptosis execution.

The NRF2/KEAP1 signaling pathway serves as a central regulatory hub of the cellular antioxidant defense system and maintains a tightly coupled upstream-downstream relationship with key biomarkers of ferroptosis. Under physiological conditions, the KEAP1 protein in the cytoplasm binds to NRF2 and promotes its ubiquitination and degradation, thereby maintaining a low basal activity of NRF2. However, under oxidative stress conditions, the inhibitory effect of KEAP1 on NRF2 is relieved. The activated NRF2 translocates into the nucleus and initiates the transcription of a series of cytoprotective genes, including SLC7A11 (the light chain of System Xc-) and HO-1. This process exerts a core inhibitory effect on ferroptosis: SLC7A11 mediates cystine uptake, which is a critical rate-limiting step for the synthesis of GSH, the essential cofactor for GPX4. GPX4 acts as the ultimate guardian against ferroptosis by reducing toxic lipid peroxides, thereby effectively blocking the execution of ferroptosis. Studies indicate that upregulation of KEAP1 expression coupled with suppression of NRF2 activity directly impairs the function of the SLC7A11-GSH-GPX4 axis. Concurrently, upregulated expression of the tumor suppressor protein P53 further transcriptionally represses SLC7A11, synergistically exacerbating lipid peroxidation accumulation and triggering ferroptosis. Conversely, activation of NRF2 enhances this antioxidant pathway and cooperatively suppresses P53, collectively boosting cellular resistance to ferroptosis. Therefore, the KEAP1/NRF2 pathway, by precisely regulating key effector molecules such as SLC7A11 and GPX4, may serve as an important modulator in the regulation of ferroptosis (Figure 9).

**FIGURE 9:**
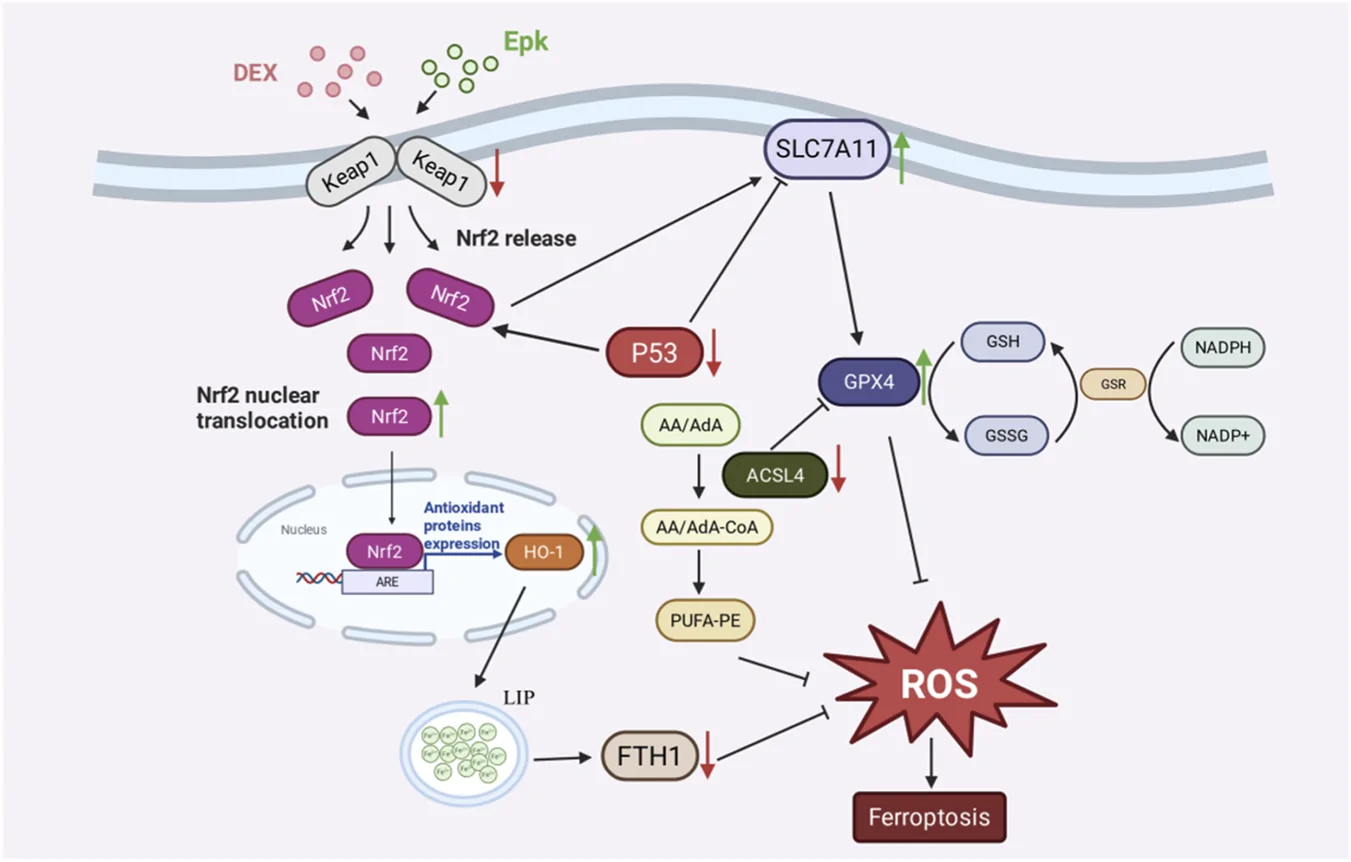
Regulatory pathways involved in ferroptosis and antioxidant response. Created in BioRender. LI, P. (2026). https://BioRender.com/0lj0aw4.

Several limitations of this study should be acknowledged. First, the conclusions regarding the KEAP1/NRF2/SLC7A11/GPX4 pathway should be interpreted with caution, as this study lacks direct causal validation experiments, including NRF2 inhibition, knockdown, overexpression, or rescue experiments, experiments examining NRF2 nuclear translocation, as well as experiments employing ferroptosis-specific inducers or inhibitors. The current findings indicate an association rather than a definitive causal mechanism. Second, although our initial focus was on osteoblastic ferroptosis, the majority of assays were performed on whole femoral tissue homogenates rather than isolated osteoblasts, and whole bone contains osteoclasts, osteocytes, marrow, and vascular/stromal cells. The lack of co-localization studies with osteoblast-specific markers and validation in primary osteoblasts or BMSC-derived osteogenic cultures limits the ability to definitively attribute the observed ferroptosis inhibition to the osteoblastic lineage; consequently, the revised title and conclusions frame our findings at the tissue level rather than as an osteoblast-specific mechanism. Third, the evidence for ferroptosis itself, while now supported by femoral 4-HNE and Fe^2+^ measurements together with changes in GPX4, SLC7A11, P53, ACSL4, FTH1, MDA, SOD, GSH/GSSG, and NADP+/NADPH, remains insufficient to establish ferroptotic cell death *per se*. More direct ferroptosis-specific evidence, such as measurement of lipid ROS (e.g., C11-BODIPY 581/591), mitochondrial morphology assessment by transmission electron microscopy, and ferroptosis-rescue experiments with ferrostatin-1/liproxstatin-1, is required. Fourth, the selection of alendronate sodium (ALN) as a positive control, while appropriate for evaluating anti-osteoporotic efficacy, lacks mechanistic specificity for the ferroptosis pathway; future studies should consider incorporating ferroptosis-specific inhibitors (e.g., Fer-1, Lip-1) or inducers (e.g., erastin) for mechanistic validation. Fifth, the rationale for selecting Epk among the four Epimedium species was based on its more pronounced efficacy observed in the initial pharmacodynamic screening; however, direct pairwise statistical comparisons among the Epimedium groups were not performed, and because all four extracts were administered at equal mass without comparative phytochemical standardization, differences in active-compound concentration may confound the interpretation that Epk is intrinsically superior. Comparative studies with chemically standardized extracts are required. Sixth, the proteomic and metabolomic analyses identified hundreds of differentially expressed molecules, yet the current study primarily focused on validating the KEAP1/NRF2/SLC7A11/GPX4 pathway; future investigations should explore additional significantly altered pathways. Seventh, Western blot, RT-qPCR, and immunofluorescence experiments were performed with six biological replicates per group (n = 6); although this is consistent with the figure legends, larger cohorts, particularly for the omics analyses, remain warranted in future studies to enhance statistical power. Finally, all experiments were conducted in animal models without *in vitro* validation in osteoblastic cell lines; cell-level studies with pathway-specific inhibitors are essential to confirm the direct effects of Epk on osteoblasts. Despite these limitations, this study provides valuable insights into the potential role of ferroptosis in GIOP and the therapeutic promise of Epk. While our rat model effectively recapitulates key features of human GIOP, species-specific differences in bone metabolism may affect the translational relevance of our findings. Additionally, the complex phytochemical composition of Epk presents challenges in identifying the specific bioactive compounds responsible for its anti-ferroptotic effects. Future studies should focus on isolating and characterizing these compounds, investigating their potential synergistic effects, and validating their efficacy in additional experimental models and ultimately in clinical settings.

In summary, this study demonstrates that Epk exerts protective effects against GIOP through preservation of bone microstructure, rebalancing of bone remodeling processes, and inhibition of ferroptosis via coordinated regulation of the KEAP1/NRF2/SLC7A11/GPX4 signaling axis. These findings advance our understanding of GIOP pathogenesis and suggest that targeting ferroptosis-related pathways may represent a promising direction for developing novel therapies in metabolic bone diseases.

## 5 Conclusion

This comprehensive study shows that Epk exerts protective effects on GIOP by preserving bone microstructure, rebalancing the bone remodeling process, and suppressing ferroptosis-associated markers in bone tissue. These effects were associated with activation of the KEAP1/NRF2 antioxidant pathway and enhancement of the SLC7A11/GPX4 signaling axis, consistent with restoration of redox homeostasis and inhibition of ferroptosis-related signaling; causal validation in cell-based and rescue models is required in future studies. These findings not only deepen our understanding of the pathogenesis of GIOP, but also provide a scientific basis for the development of new treatments for metabolic bone diseases targeting ferroptosis. Epk’s multi-target strategy is promising and merits further clinical investigation.

## Data Availability

The proteomics data have been submitted to the ProteomeXchange Consortium via the PRIDE repository, accession number PXD084776.
