# Supplementary material for: Epimedium koreanum Nakai attenuates glucocorticoid-induced osteoporosis by suppressing ferroptosis via the KEAP1/NRF2/SLC7A11/GPX4 axis: comparative pharmacodynamic evaluation of four epimedium species and proteomic and metabolomic analyses of epk
Source: Front Pharmacol. 2026 Oct 29;17:1948777. doi: 10.3389/fphar.2026.1948777 (PMC13754854; doi:10.3389/fphar.2026.1948777)
Supplement: Supplementary file 1 [file Supplementaryfile1.pdf]

S1. Preparation and chemical standardization of the Epimedium extracts

Supplementary Methods

S1.1 Botanical materials. The four Epimedium raw materials were purchased from their main production areas: Epb (Batch No. TA220201001) from Lixian County, Gansu Province; Eps (Batch No. TA220250001) from Fengdu County, Chongqing; Epk (Batch No. TA220571001) from Tonghua City, Jilin Province; and Epp (Batch No. TA220252001) from Dianjiang County, Chongqing. The botanical authentication was performed by Associate Professor Guangzhi Cai (Changchun University of Chinese Medicine), and the four materials were identified as the dried leaves of *Epimedium brevicornu* Maxim. (Epb), *Epimedium sagittatum* (Sieb. et Zucc.) Maxim. (Eps), *Epimedium koreanum* Nakai (Epk) and *Epimedium pubescens* Maxim. (Epp) (family Berberidaceae). The raw materials were authenticated after purchase, and no voucher specimens were deposited in a herbarium.

S1.2 Preparation of the Epimedium extracts

The dried leaves of each Epimedium species were pulverized and passed through a No. 3 sieve. Approximately 42 g of the powder was accurately weighed into a stoppered conical flask, 200 mL of 70% ethanol was accurately added, and the mixture was sonicated (400 W, 50 kHz) for 1 h. The ethanol was removed by rotary evaporation until free of alcohol odour, and the residue was adjusted to 200 mL with purified water to obtain the extract. The extracts were stored at −20 °C until use.

S1.3 Preparation of the mixed reference standard solution

Accurately weighed reference standards of epimedin A, epimedin B, epimedin C and icariin (10 mg of each) were dissolved in methanol and diluted to 10 mL in volumetric flasks to obtain a mixed standard solution at 0.10 mg/mL.

S1.4 Sample preparation

The extract obtained under S1.2 was filtered, and the successive filtrate was collected for the determination of flavonol glycosides.

S1.5 HPLC conditions

The flavonoids were determined by the HPLC method previously established in our group. A Diamonsil Plus C18 column (5 μm, 250 mm × 4.6 mm) was used; mobile

phase A was acetonitrile and mobile phase B was water; the detection wavelength was 270 nm, the column temperature was 35 °C, and the injection volume was 10 μL. The gradient elution program is shown in Table S1.

Table S1. HPLC gradient elution program.

Time (min)	Mobile phase A (%)	Mobile phase B (%)
0-29	24	76
29-35	24→42	76→58
35-55	42	58
55-65	24	76

S1.6 Content determination of flavonol glycosides

According to the assay requirement of the Chinese Pharmacopoeia (2020 edition) for Epimedium, the total content of epimedin A, epimedin B, epimedin C and icariin, calculated on the dried material, should be not less than 0.50% for Epimedium koreanum and not less than 1.5% for Epimedium brevicornu, Epimedium sagittatum and Epimedium pubescens. The flavonol glycosides of the four species were determined by HPLC; the results are shown in Table S2 and the representative chromatograms are shown in Fig. S1.

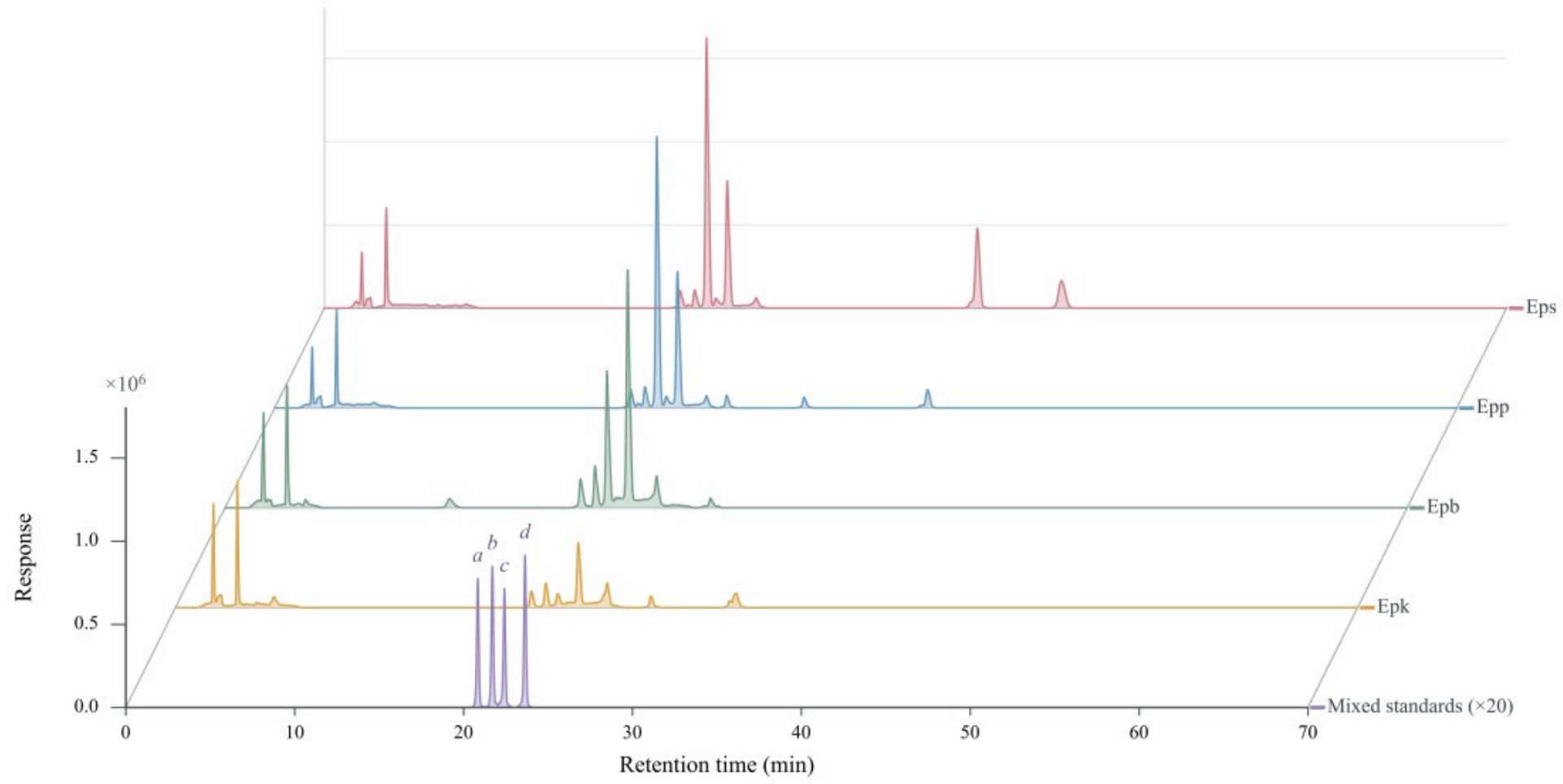

Fig. S1. HPLC chromatograms of the four Epimedium extracts. a, epimedin A; b, epimedin B; c, epimedin C; d, icariin.

Table S2. Content determination of flavonol glycosides in the four Epimedium extracts (%, calculated on dried material).

Species	Content（%）					Conclusion
	Epimedin A	Epimedin B	Epimedin C	Icariin	Total	
Epimedium koreanum	0.18	0.25	0.35	0.64	1.43	Meets the requirement
Epimedium brevicornu	0.40	0.51	4.00	2.85	7.77	Meets the requirement
Epimedium pubescens	0.25	0.26	7.25	1.50	9.27	Meets the requirement
Epimedium sagittatum	0.23	0.23	7.27	1.41	9.14	Meets the requirement

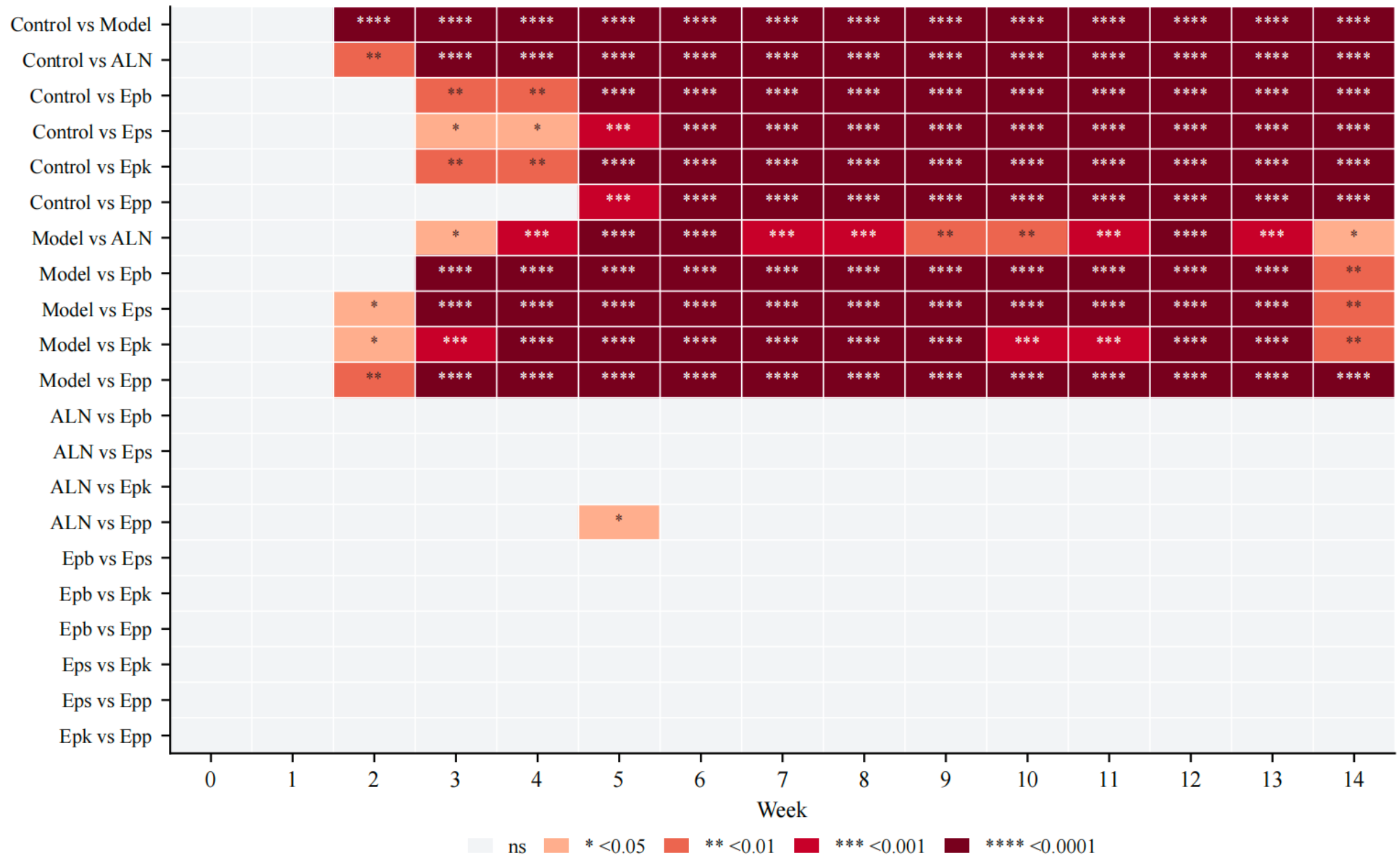

Fig. S2. All pairwise comparisons of body weight between groups at each week (two-way repeated-measures ANOVA with Šídák's multiple-comparison test). *P < 0.05, **P < 0.01, ***P < 0.001, ****P < 0.0001; ns, not significant.

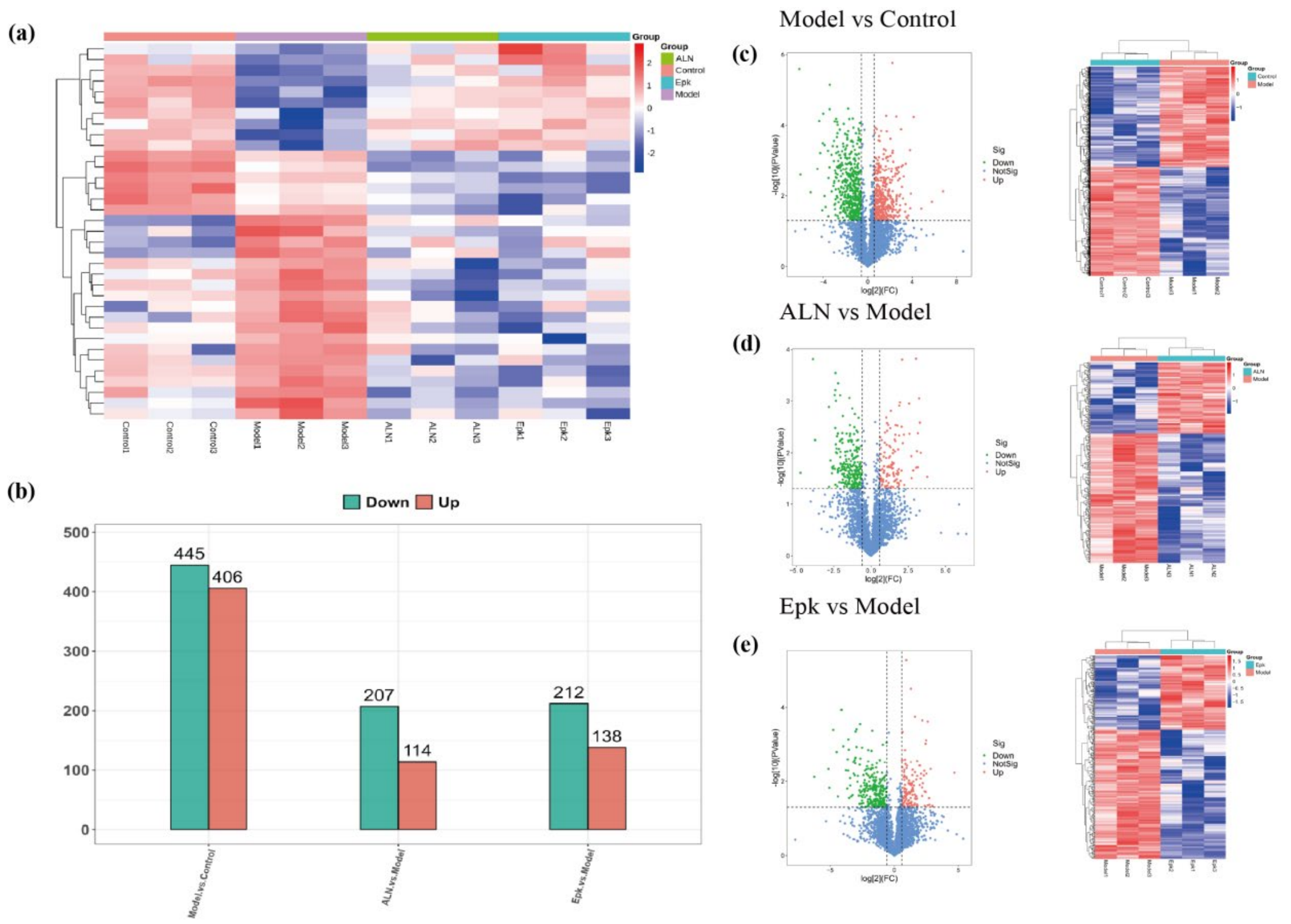

Fig. S3. Proteomic analysis reveals functional alterations in GIOP rats and response to Epk treatment. (a) IAll Sample Cluster Analysis Diagram. (b-e) Intergroup Difference Protein Bar Charts, Volcano Plots, and Cluster Analysis Diagrams.

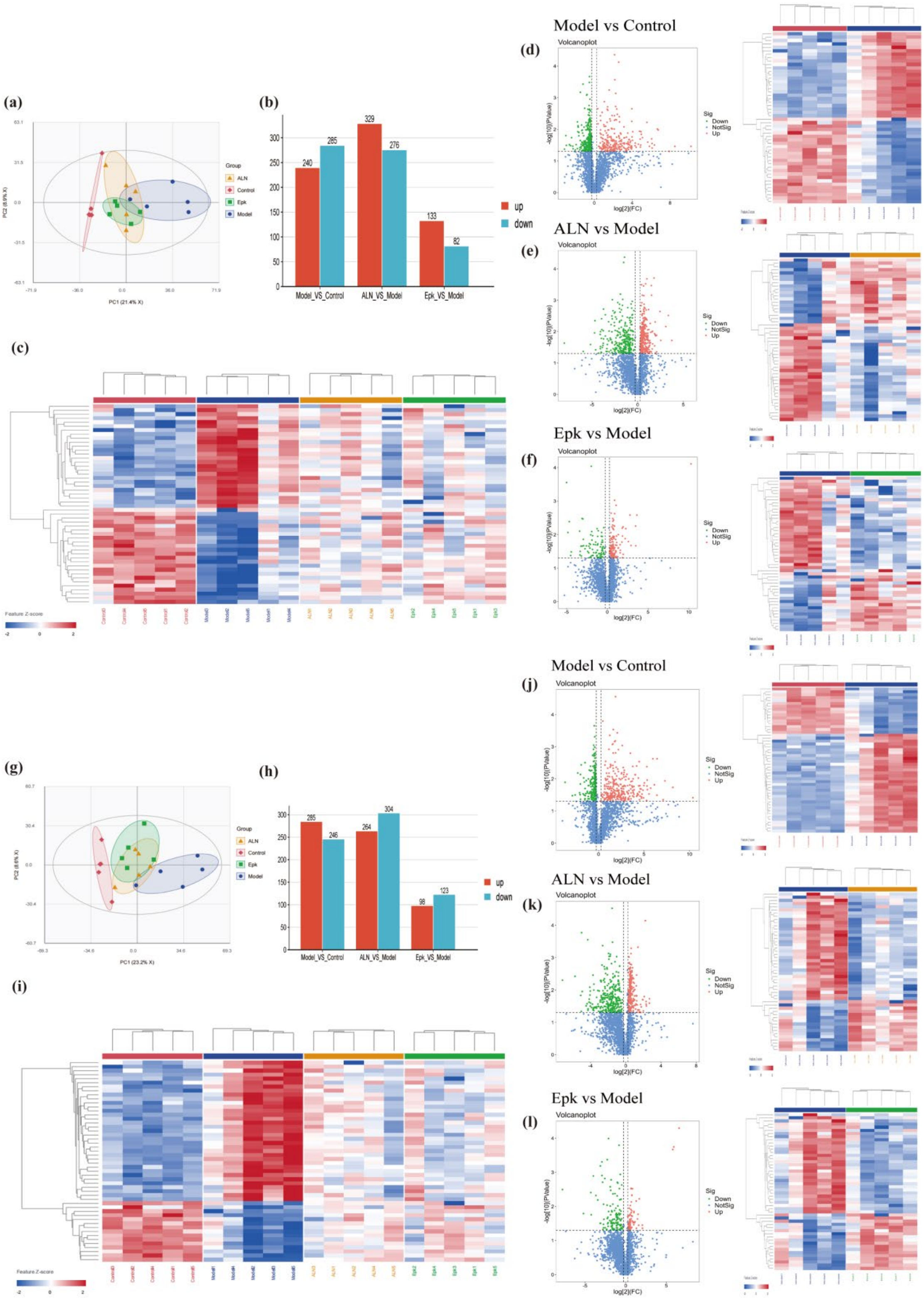

Fig. S4. Epk primarily exerts its GIOP-delaying effects through lipid metabolism and amino acid metabolism pathways. (a-f) Positive Ion Mode (ESI+) Intergroup Differential Metabolite PCA Score Plot, Bar Chart, Volcano Plot, and Cluster Analysis Diagram. (g-i) Negative Ion Mode (ESI-) Intergroup Differential Metabolites PCA Score Plot, Bar Chart, Volcano Plot, and Cluster Analysis Diagram.

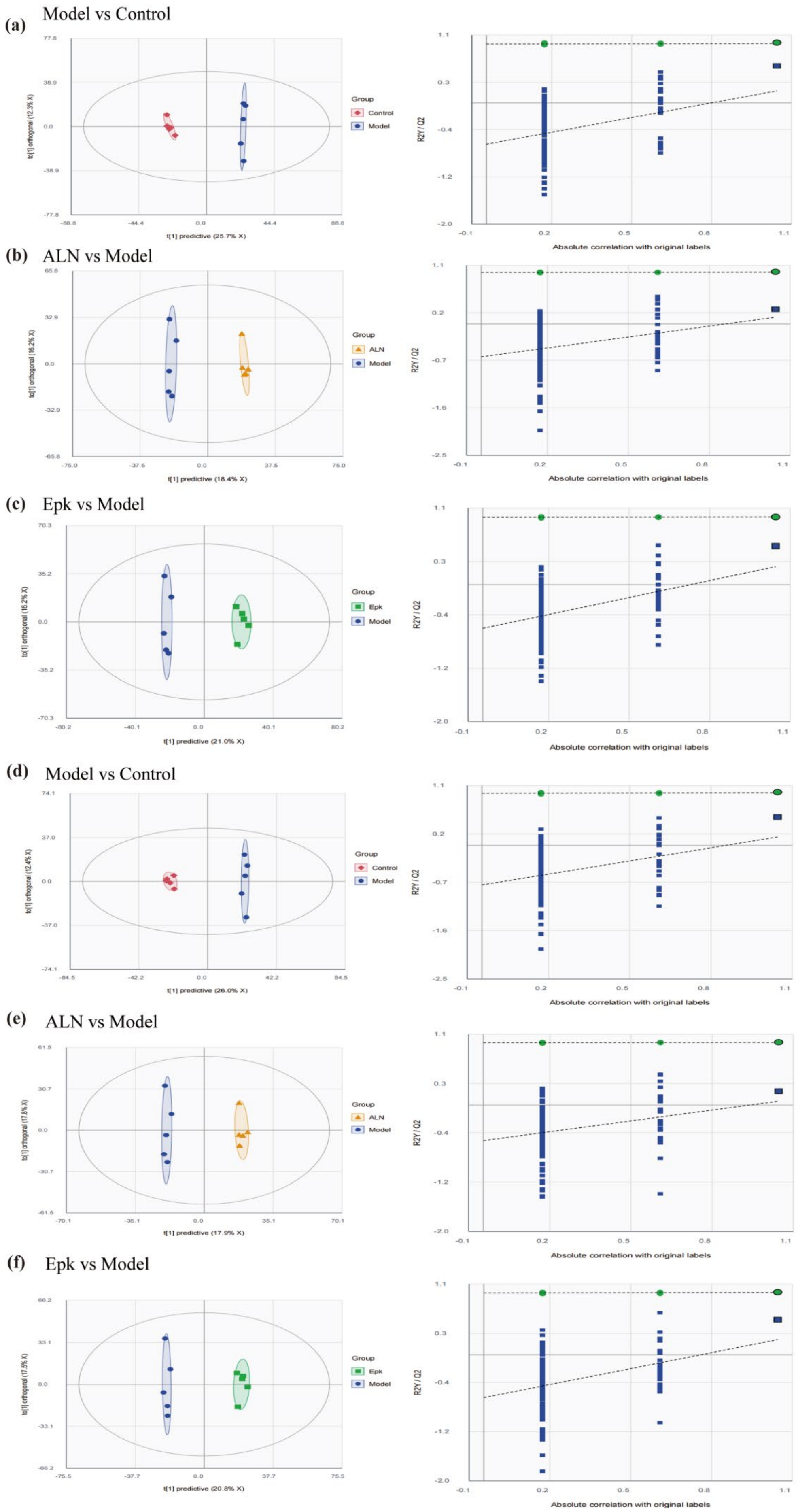
(a) Model vs Control
Group
Control
Model
to[1] orthogonal (12.3% X)
t[1] predictive (25.7% X)
R2Y / Q2
Absolute correlation with original labels
(b) ALN vs Model
Group
ALN
Model
to[1] orthogonal (16.2% X)
t[1] predictive (18.4% X)
R2Y / Q2
Absolute correlation with original labels
(c) Epk vs Model
Group
Epk
Model
to[1] orthogonal (16.2% X)
t[1] predictive (21.0% X)
R2Y / Q2
Absolute correlation with original labels
(d) Model vs Control
Group
Control
Model
to[1] orthogonal (12.4% X)
t[1] predictive (26.0% X)
R2Y / Q2
Absolute correlation with original labels
(e) ALN vs Model
Group
ALN
Model
to[1] orthogonal (17.8% X)
t[1] predictive (17.9% X)
R2Y / Q2
Absolute correlation with original labels
(f) Epk vs Model
Group
Epk
Model
to[1] orthogonal (17.5% X)
t[1] predictive (20.8% X)
R2Y / Q2
Absolute correlation with original labels

Fig. S5. OPLS-DA analysis and validation of intergroup differential metabolites. (a–c) OPLS-DA score plots of differential metabolites among groups in positive ion mode (ESI+). (d–f) OPLS-DA score plots of differential metabolites among groups in negative ion mode (ESI−). Model robustness was evaluated by 200× permutation testing, and the corresponding R^2 and Q^2 values are reported in Table S3.

Table S3. Results of the 200× permutation tests for the OPLS-DA models, with the corresponding R^2 and Q^2 values.

Pattern	Group comparison	R^2X	R^2Y	Q^2	Permutation p （Q^2）	Permutation p （R^2Y）
ESI−	Model vs Control	0.3839	0.9946	0.5289	0.00498	0.05473
ESI−	ALN vs Model	0.3577	0.9935	0.2192	0.04975	0.17413
ESI−	Epk vs Model	0.3825	0.9927	0.5540	0.01493	0.13930
ESI+	Model vs Control	0.3797	0.9958	0.6109	0.00498	0.07463
ESI+	ALN vs Model	0.3454	0.9949	0.2813	0.03483	0.12935
ESI+	Epk vs Model	0.3717	0.9900	0.5636	0.01493	0.35821

Table S4 Differential metabolites

NO.	Ret Time	Measured *m/z*	metabolite	Adduct	Formula	Intergroup comparison		
						Model vs Control	ALN vs Model	Epk vs Model
1	0.7551	459.1508	Methyl salicylate O-[rhamnosyl-(1->6)-glucoside]	$[M-H]^-$	$C_{20}H_{28}O_{12}$	Down	Up	Up
2	0.7853	192.0478	Brassicanal A	$[M+H]^+$	$C_{10}H_9NOS$	Down	Up	Up
3	0.8166	260.0530	Glucosamine 6-phosphate	$[M+H]^+$	$C_6H_{14}NO_8P$	Up	ns	ns
4	0.8395	259.0221	Fructose 6-phosphate	$[M-H]^-$	$C_6H_{13}O_9P$	Up	ns	ns
5	0.8480	173.0210	Glycerol 3-phosphate	$[M+H]^+$	$C_3H_9O_6P$	Up	ns	ns
6	0.8637	291.1294	Argininosuccinic acid	$[M+H]^+$	$C_{10}H_{18}N_4O_6$	Up	ns	ns
7	0.8658	300.0484	N-Acetyl-D-Glucosamine 6-Phosphate	$[M-H]^-$	$C_8H_{16}NO_9P$	Up	ns	ns
8	0.9028	114.0197	4-Oxo-2-azetidinecarboxylic acid	$[M-H]^-$	$C_4H_5NO_3$	Down	Up	Up
9	0.9028	238.0946	Dihydrobiopterin	$[M-H]^-$	$C_9H_{13}N_5O_3$	Down	ns	ns
10	0.9081	221.0594	L-Cystathionine	$[M-H]^-$	$C_7H_{14}N_2O_4S$	Down	ns	ns
11	0.9158	248.1489	3-Hydroxybutyrylcarnitine	$[M+H]^+$	$C_{11}H_{21}NO_5$	Down	Up	Up
12	0.9187	665.2146	Glycogen	$[M-H]^-$	$C_{24}H_{42}O_{21}$	Up	ns	ns
13	1.5753	307.0347	dUMP	$[M-H]^-$	$C_9H_{13}N_2O_8P$	Up	Down	Down
14	1.9246	123.0555	Niacinamide	$[M+H]^+$	$C_6H_6N_2O$	Up	Down	ns
15	2.3080	68.9958	Trifluoromethane	$[M-H]^-$	CHF_3	Up	Down	Down
16	2.3596	189.0069	6-Chloro-2-pyridylmethyl nitrate	$[M+H]^+$	$C_6H_5ClN_2O_3$	Down	Up	Up
17	2.6503	483.0477	Iodiconazole	$[M-H]^-$	$C_{19}H_{19}F_2IN_4O$	Down	Up	Up
18	2.7775	274.0224	Meticrane	$[M-H]^-$	$C_{10}H_{13}NO_4S_2$	Down	Up	Up

Table S4 (Continued) Differential metabolites

NO.	Ret Time	Measured *m/z*	metabolite	Adduct	Formula	Intergroup comparison		
						Model vs Control	ALN vs Model	Epk vs Model
19	2.7839	230.0129	2,5-Dioxopyrrolidin-1-yl 2-(acetylthio)acetate	[M-H]-	$C_8H_9NO_5S$	Down	Up	Up
20	2.7922	386.2286	L-Proline, 1-(N-((3-((propylamino)carbonyl)oxiranyl)carbonyl)-L-isoleucyl)-, (2S-trans)-	$[M+H]^+$	$C_{18}H_{31}N_3O_6$	Up	Down	Down
21	2.8840	218.0659	O-Succinyl-L-homoserine	$[M-H]^-$	$C_8H_{13}NO_6$	Up	Down	Down
22	3.2003	315.0726	Ginnalin B	$[M-H]^-$	$C_{13}H_{16}O_9$	Up	Down	Down
23	3.2003	279.1027	Phenprocoumon	$[M-H]^-$	$C_{18}H_{16}O_3$	Up	Down	Down
24	3.2420	179.9840	Fluoranil	$[M]^-$	$C_6F_4O_2$	Down	Up	Up
25	3.3760	302.1983	1-[(5-Methoxy-3-indolylidene)methylamino]-2-pentylguanidine	$[M+H]^+$	$C_{16}H_{23}N_5O$	Up	Down	Down
26	3.5150	243.0975	Thymidine	$[M+H]^+$	$C_{10}H_{14}N_2O_5$	Down	Up	Up
27	3.5711	243.0440	(5R,6S)-3-(Aminomethyl)-6-[(1R)-1-hydroxyethyl]-7-oxo-4-thia-1-azabicyclo[3.2.0]hept-2-ene-2-carboxylic acid	$[M-H]^-$	$C_9H_{12}N_2O_4S$	Up	Down	Down
28	3.6219	184.9856	3-Phosphoglyceric acid	$[M-H]^-$	$C_3H_7O_7P$	Up	ns	ns
29	3.7517	410.2261	BUTAPERAZINE	$[M+H]^+$	$C_{24}H_{31}N_3OS$	Up	Down	Down
30	3.8120	140.0466	Dimetridazole	$[M-H]^-$	$C_5H_7N_3O_2$	Up	Down	Down
31	3.8120	209.0819	6-(4-methyl-2-oxopentyl)-4-hydroxy-2-pyrone	$[M-H]^-$	$C_{11}H_{14}O_4$	Up	Down	Down

Table S4 (Continued) Differential metabolites

NO.	Ret Time	Measured *m/z*	metabolite	Adduct	Formula	Intergroup comparison		
						Model vs Control	ALN vs Model	Epk vs Model
32	3.8120	218.1025	Pantothenic acid	$[M-H]^-$	$C_9H_{17}NO_5$	Up	ns	Down
33	3.8403	206.1386	Panthenol	$[M+H]^+$	$C_9H_{19}NO_4$	Down	Up	Up
34	3.9069	529.2632	Cinncassiol D2 glucoside	$[M-H]^-$	$C_{26}H_{42}O_{11}$	Up	Down	Down
35	3.9422	403.1842	Tetraxetan	$[M-H]^-$	$C_{16}H_{28}N_4O_8$	Up	Down	Down
36	4.1114	239.1485	Pentaethylene glycol	$[M+H]^+$	$C_{10}H_{22}O_6$	Down	Up	Up
37	4.1532	867.4707	Lc3Cer	$[M-H]^-$	$C_{40}H_{72}N_2O_{18}$	Down	Down	Down
38	4.2842	363.2161	Cortisol	$[M+H]^+$	$C_{21}H_{30}O_5$	Up	n	ns
39	4.3029	465.2130	T-2 toxin	$[M-H]^-$	$C_{24}H_{34}O_9$	Up	Down	Down
40	4.3161	561.2532	Protoporphyrin IX	$[M-H]^-$	$C_{34}H_{34}N_4O_4$	Up	Down	ns
41	4.4106	229.0176	3-Phenylpropionic acid sulfate	$[M-H]^-$	$C_9H_{10}O_5S$	Up	Down	Down
42	4.7229	551.3046	Arbekacin	$[M-H]^-$	$C_{22}H_{44}N_6O_{10}$	Up	Down	Down
43	4.7964	234.9836	Disodium hydrogen citrate	$[M-H]^-$	$C_6H_6Na_2O_7$	Down	Up	Up
44	4.9551	532.2737	Azeliragon	$[M+H]^+$	$C_{32}H_{38}ClN_3O_2$	Up	Down	Down
45	5.0081	495.2963	Pregnanediol 3-O-glucuronide	$[M-H]^-$	$C_{27}H_{44}O_8$	Up	Down	Down
46	5.0409	438.1268	Sporamin	$[M-H]^-$	$C_{21}H_{30}BrNO_4$	Up	Down	Down
47	5.0409	205.0506	Scoparone	$[M-H]^-$	$C_{11}H_{10}O_4$	Up	Down	Down
48	5.1765	137.0009	Fosfomycin	$[M-H]^-$	$C_3H_7O_4P$	Down	Up	Up
49	5.2407	86.0070	thiazoline	$[M-H]^-$	C_3H_5NS	Down	Up	Up
50	5.2598	595.2558	Chlorin E6	$[M-H]^-$	$C_{34}H_{36}N_4O_6$	Up	Down	Down
51	5.5820	516.3007	Taurocholic acid	$[M+H]^+$	$C_{26}H_{45}NO_7S$	Up	ns	ns
52	5.6044	199.0962	Guaifenesin	$[M+H]^+$	$C_{10}H_{14}O_4$	Down	Up	Up

Table S4 (Continued) Differential metabolites

NO.	Ret Time	Measured *m/z*	metabolite	Adduct	Formula	Intergroup comparison		
						Model vs Control	ALN vs Model	Epk vs Model
53	5.6374	346.1261	Dehydromonocrotaline	$[M+Na]^+$	$C_{16}H_{21}NO_6$	Down	Down	Down
54	5.6694	170.9856	5-Chlorosalicylic acid	$[M-H]^-$	$C_7H_5ClO_3$	Down	Up	Up
55	5.6694	142.9905	4-Chlorocatechol	$[M-H]^-$	$C_6H_5ClO_2$	Down	Up	Up
56	5.7195	207.0663	(R)-2-Benzylsuccinate	$[M-H]^-$	$C_{11}H_{12}O_4$	Down	Up	Up
57	5.7738	275.1390	indole-3-acetyl-valine	$[M+H]^+$	$C_{15}H_{18}N_2O_3$	Up	Down	Down
58	5.8372	196.9828	1-Iodopentane	$[M-H]^-$	$C_5H_{11}I$	Down	Up	Up
59	5.8763	715.3555	Cheirotoxol	$[M+H]^+$	$C_{35}H_{54}O_{15}$	Up	Down	Down
60	5.8989	297.0978	2-Phenylethanol glucuronide	$[M-H]^-$	$C_{14}H_{18}O_7$	Up	Down	Down
61	5.9929	697.3794	Periplocin	$[M+H]^+$	$C_{36}H_{56}O_{13}$	Up	Down	Down
62	6.0945	200.9967	Benzoyl phosphate	$[M-H]^-$	$C_7H_7O_5P$	Down	Up	Up
63	6.1551	185.0004	1-Fluoro-2,4-dinitrobenzene	$[M-H]^-$	$C_6H_3FN_2O_4$	Down	Up	Up
64	6.4094	206.9873	2-Nitrobenzoic acid thiocyanate	$[M-H]^-$	$C_8H_4N_2O_3S$	Down	Up	Up
65	6.4436	277.1394	Saccharopine	$[M+H]^+$	$C_{11}H_{20}N_2O_6$	Down	Up	ns
66	6.5262	157.0060	Phenylphosphonic acid	$[M-H]^-$	$C_6H_7O_3P$	Down	Up	Up
67	6.5553	115.0084	3-Amino-5-mercapto-1,2,4-triazole	$[M-H]^-$	$C_2H_4N_4S$	Down	Up	Up
68	6.5757	807.7436	TG(16:0/16:0/16:0)	$[M+H]^+$	$C_{51}H_{98}O_6$	Up	ns	ns
69	6.7282	218.9862	Phosphoramide mustard	$[M-H]^-$	$C_4H_{11}C_{l2}N_2O_2P$	Down	Up	Up
70	6.7681	211.0175	Zonisamide	$[M-H]^-$	$C_8H_8N_2O_3S$	Down	Up	Up
71	6.7966	223.0401	1-Hydroxyanthraquinone	$[M-H]^-$	$C_{14}H_8O_3$	Up	Down	Down
72	6.8816	223.0166	1-Naphthyl phosphate	$[M-H]^-$	$C_{10}H_9O_4P$	Down	Up	Up

Table S4 (Continued) Differential metabolites

NO.	Ret Time	Measured *m/z*	metabolite	Adduct	Formula	Intergroup comparison Model vs Control	ALN vs Model	Epk vs Model
73	6.9153	472.2372	(2R)-5-(Diaminomethylideneamino)-2-[[2,2-di(phenyl)acetyl]amino]-N-[(4-hydroxyphenyl)methyl]pentanamide	$[M-H]^-$	$C_{27}H_{31}N_5O_3$	Up	Down	Down
74	7.0609	151.0084	Mercaptopurine	$[M-H]^-$	$C_5H_4N_4S$	Down	Up	Up
75	7.0897	161.0592	Naphthalene-1,2-diol	$[M+H]^+$	$C_{10}H_8O_2$	Down	Up	Up
76	7.1774	300.9914	Erythrityl Tetranitrate	$[M-H]^-$	$C_4H_6N_4O_{12}$	Down	Up	Up
77	7.1829	169.0329	2,5-Dimethyl-3-furanthiol acetate	$[M-H]^-$	$C_8H_{10}O_2S$	Down	Up	Up
78	7.1836	341.0881	Aesculin	$[M+H]^+$	$C_{15}H_{16}O_9$	Down	Up	Up
79	7.2548	168.9907	Glyceraldehyde 3-phosphate	$[M-H]^-$	$C_3H_7O_6P$	Down	Up	Up
80	7.3638	329.0035	Pyrroloquinoline quinone	$[M-H]^-$	$C_{14}H_6N_2O_8$	Down	Up	ns
81	7.3745	381.0351	Mefruside	$[M-H]^-$	$C_{13}H_{19}ClN_2O_5S_2$	Down	Up	Up
82	7.3853	171.0064	Glycerol 3-phosphate	$[M-H]^-$	$C_3H_9O_6P$	Down	Up	ns
83	7.3960	306.9989	(R)-5-Diphosphomevalonic acid	$[M-H]^-$	$C_6H_{14}O_{10}P_2$	Down	Up	ns
84	7.4123	220.9809	Acetazolamide	$[M-H]^-$	$C_4H_6N_4O_3S_2$	Down	Up	Up
85	7.4151	217.0789	1-Benzyl-4-(chloromethyl)benzene	$[M+H]^+$	$C_{14}H_{13}Cl$	Down	Up	Up
86	7.4257	353.2310	Prostaglandin I2	$[M+H]^+$	$C_{20}H_{32}O_5$	Down	ns	ns
87	7.4618	150.9893	Oxidized dithiothreitol	$[M-H]^-$	$C_4H_8O_2S_2$	Down	Up	Up
88	7.4888	409.0105	Butoconazole	$[M-H]^-$	$C_{19}H_{17}Cl_3N_2S$	Down	Up	Up

Table S4 (Continued) Differential metabolites

NO.	Ret Time	Measured *m/z*	metabolite	Adduct	Formula	Intergroup comparison		
						Model vs Control	ALN vs Model	Epk vs Model
89	7.4888	323.0108	2'-Deoxy-5'-O-thiophosphonouridine	$[M-H]^-$	$C_9H_{13}N_2O_7PS$	Down	Up	Up
90	7.6097	206.9978	(Z)-[3-(Methylsulfinyl)-1-propenyl] 2-propenyl disulfide	$[M-H]^-$	$C_7H_{12}OS_3$	Down	Up	Up
91	7.6808	137.9906	2-C-methyl-D-erythritol-2,4-cyclodi phosphate	$[M]^{2-}$	$C_5H_{10}O_9P_2^{2-}$	Down	Up	Up
92	7.8196	265.1331	4(3H)-Quinazolinone, 2-ethyl-3-(o-tolyl)-	$[M+H]^+$	$C_{17}H_{16}N_2O$	Down	Up	Up
93	8.0027	385.1769	Olodaterol	$[M-H]^-$	$C_{21}H_{26}N_2O_5$	Down	Down	Down
94	8.0063	387.1921	Olodaterol	$[M+H]^+$	$C_{21}H_{26}N_2O_5$	Down	Down	Down
95	8.0282	272.2577	Hexadecasphingosine	$[M+H]^+$	$C_{16}H_{33}NO_2$	Down	Up	Up
96	8.1180	493.3401	Sphingomyelins	$[M]^+$	$C_{24}H_{50}N_2O_6P^+$	Down	Down	Down
97	8.1199	173.1536	Capric acid	$[M+H]^+$	$C_{10}H_{20}O_2$	Down	Up	ns
98	8.1683	135.0803	7-Nonene-3,5-diyn-1-ol	$[M+H]^+$	$C_9H_{10}O$	Down	Up	Up
99	8.1736	327.0773	TRIPHENYL PHOSPHATE	$[M+H]^+$	$C_{18}H_{15}O_4P$	Down	Up	Up
100	8.2004	328.2857	Dihydroceramide	$[M-H]^-$	$C_{19}H_{39}NO_3$	Up	ns	ns
101	8.2936	233.1538	Valerenic acid	$[M-H]^-$	$C_{15}H_{22}O_2$	Down	Up	Up
102	8.3380	205.1584	(6R,7S)-6,7-Epoxy-1,3-tetradecadiyn e	$[M+H]^+$	$C_{14}H_{20}O$	Down	Up	Up
103	8.3481	201.1649	(-)-alpha-Curcumene	$[M-H]^-$	$C_{15}H_{22}$	Down	Up	Up
104	8.3754	227.1139	Carnosine	$[M+H]^+$	$C_9H_{14}N_4O_3$	Down	Up	Up
105	8.4021	185.0444	3,4-Dihydroxymandelic acid	$[M+H]^+$	$C_8H_8O_5$	Down	Up	Up

Table S4 (Continued) Differential metabolites

NO.	Ret Time	Measured *m/z*	metabolite	Adduct	Formula	Intergroup comparison		
						Model vs Control	ALN vs Model	Epk vs Model
106	8.4765	504.4037	Tetracosatetraenoyl carnitine	$[M+H]^+$	$C_{31}H_{53}NO_4$	Down	Down	Down
107	8.6140	203.9447	Chlorthiamid	$[M-H]^-$	$C_7H_5Cl_2NS$	Down	Up	Up
108	8.6140	160.9334	1-Propanol, 3,3,3-trichloro-	$[M-H]^-$	$C_3H_5Cl_3O$	Down	Up	Up
109	8.6140	142.9226	1,1,2-Trichloropropene	$[M-H]^-$	$C_3H_3Cl_3$	Down	Up	Up
110	8.7732	332.1969	5-hydroxy saxagliptin	$[M+H]^+$	$C_{18}H_{25}N_3O_3$	Down	Up	Up
111	8.8187	367.1147	trans-3-Hydroxycotinine glucuronide	$[M-H]^-$	$C_{16}H_{20}N_2O_8$	Down	Up	Up
112	8.9955	443.3003	Ascorbyl stearate	$[M+H]^+$	$C_{24}H_{42}O_7$	Up	Down	Down
113	9.2279	392.2919	N-Palmitoyl Histidine	$[M-H]^-$	$C_{22}H_{39}N_3O_3$	Down	Up	Up
114	9.2890	468.3333	CCR4 Antagonist	$[M+H]^+$	$C_{27}H_{41}N_5O_2$	Down	Up	Up
115	9.3151	338.1972	3,6-Ditigloyloxytropan-7-ol	$[M+H]^+$	$C_{18}H_{27}NO_5$	Down	Up	Up
116	9.3774	151.0434	1-Pentanesulfonic acid	$[M-H]^-$	$C_5H_{12}O_3S$	Down	Up	Up
117	9.3827	303.2326	Eicosatetraenoic acid	$[M-H]^-$	$C_{20}H_{32}O_2$	Down	Up	Up
118	9.4460	477.3211	Polyporusterone B	$[M+H]^+$	$C_{28}H_{44}O_6$	Down	Up	Up
119	9.4513	472.0762	Clamikalant	$[M+H]^+$	$C_{19}H_{22}ClN_3O_5S_2$	Down	Up	Up
120	9.5622	355.1580	6-Amino-4-(4-phenoxyphenylet hylamino)quinazoline	$[M-H]^-$	$C_{22}H_{20}N_4O$	Down	Up	Up
121	9.8929	563.2644	Protoporphyrin IX	$[M+H]^+$	$C_{34}H_{34}N_4O_4$	Up	ns	ns
122	9.9623	203.1291	(R)-6-(3-Methylpiperazin-1-yl)n icotinonitrile	$[M+H]^+$	$C_{11}H_{14}N_4$	Down	Up	Up
123	10.0520	211.0999	2-Ethyl-1-hexanol sulfate	$[M+H]^+$	$C_8H_{18}O_4S$	Down	Up	Up

Table S4 (Continued) Differential metabolites

NO.	Ret Time	Measured *m/z*	metabolite	Adduct	Formula	Intergroup comparison		
						Model vs Control	ALN vs Model	Epk vs Model
124	10.0531	209.0853	2-Ethyl-1-hexanol sulfate	$[M-H]^-$	$C_8H_{18}O_4S$	Down	Up	Up
125	10.1064	213.0692	1,2,3,4-Tetrahydroisoquinoline-7-sulfonamide	$[M+H]^+$	$C_9H_{12}N_2O_2S$	Down	Up	Up
126	10.1388	61.9884	Nitrate	$[M]^-$	NO_3^-	Down	Up	Up
127	10.3569	419.3520	7-a,25-Dihydroxycholesterol	$[M+H]^+$	$C_{27}H_{46}O_3$	Down	Up	Up
128	10.3895	701.4378	PA(14:0/20:4(8Z,11Z,14Z,17Z)-2OH(5S,6R))	$[M+H]^+$	$C_{37}H_{65}O_{10}P$	Down	Up	Up
129	10.6348	499.3993	TG(10:0/8:0/8:0)	$[M+H]^+$	$C_{29}H_{54}O_6$	Down	Up	Up
130	10.6945	427.3782	Dioctyl sebacate	$[M+H]^+$	$C_{26}H_{50}O_4$	Down	Up	Up
131	10.7323	221.0315	Imidazole acetol-phosphate	$[M+H]^+$	$C_6H_9N_2O_5P$	Down	Up	Up
132	10.8803	396.2636	N-Tert-Butyl-3-(4-(2-methoxyphenyl)-piperazin-1-yl)-2-phenyl propanamide	$[M+H]^+$	$C_{24}H_{33}N_3O_2$	Down	Up	Up
133	10.8855	701.5603	SM(d18:1/16:0)	$[M-H]^-$	$C_{39}H_{79}N_2O_6P$	Down	Up	Up
134	12.1488	564.5361	Cer(d18:1/18:0)	$[M-H]^-$	$C_{36}H_{71}NO_3$	Down	Up	ns
135	13.3965	833.7593	TG(16:0/16:0/18:1(9Z))	$[M+H]^+$	$C_{53}H_{100}O_6$	Down	Up	Up
136	13.7565	416.2196	Estradiol 17-dihydrotrigonelline	$[M+Na]^+$	$C_{25}H_{31}NO_3$	Down	Up	Up
137	13.8006	395.3308	(3beta,22E,24R)-Ergosta-4,6,8(14),22-tetraen-3-ol	$[M+H]^+$	$C_{28}H_{42}O$	Down	Up	Up
138	13.9941	577.5185	Cohibin C	$[M+H]^+$	$C_{37}H_{68}O_4$	Down	Up	Up
139	14.0217	595.5296	DG(16:0/18:1(11Z)/0:0)	$[M+H]^+$	$C_{37}H_{70}O_5$	Down	Up	Up

Table S4 (Continued) Differential metabolites

NO.	Ret Time	Measured *m/z*	metabolite	Adduct	Formula	Intergroup comparison		
						Model vs Control	ALN vs Model	Epk vs Model
140	14.1266	727.6214	DG(20:3(5Z,8Z,11Z)/24:1(15Z)/0:0)[(2S)-1-hydroxy-3-[(5Z,8Z,11Z)-icosa-5,8,11-trienoyl]oxypropan-2-yl] (Z)-tetracos-15-enoate	$[M-H]^-$	$C_{47}H_{84}O_5$	Down	Up	Up
141	14.2895	108.0203	Imidazooxazole	$[M-H]^-$	$C_4H_3N_3O$	Down	Up	Up
142	14.9374	110.9675	Methylphosphonothiolate	$[M-H]^-$	CH_5O_2PS	Down	Up	Up
143	14.9433	207.2107	Cadinene, (-)-	$[M+H]^+$	$C_{15}H_{26}$	Down	Up	Up
144	14.9610	135.0811	4-Propylphenol	$[M-H]^-$	$C_9H_{12}O$	Down	Up	Up
145	15.0789	161.0242	Umbelliferone	$[M-H]^-$	$C_9H_6O_3$	Down	Up	Up
146	15.0846	242.8948	1,2,3,5-Tetrachloro-4-methoxybenzene	$[M-H]^-$	$C_7H_4Cl_4O$	Down	Up	Up
147	15.1157	175.0502	2-Quinoxalinecarboxylic acid	$[M+H]^+$	$C_9H_6N_2O_2$	Down	Up	Up
148	15.1294	149.9910	Hexafluoropropene	$[M]^-$	C_3F_6	Down	Up	Up
149	15.1967	126.9050	Iodide	$[M]^-$	I^-	Down	Up	Up
150	15.2214	272.9595	Ribose-1-arsenate	$[M-H]^-$	$C_5H_{11}AsO_8$	Down	Up	ns
151	15.2214	80.9652	Hydrogen sulfite (bisulfite)	$[M]^-$	HSO_3^-	Down	Up	ns
152	15.3283	229.8736	2,3,5,6-Tetrachloro-4-pyridinol	$[M-H]^-$	C_5HCl_4NO	Down	Up	Up
153	15.9928	199.0600	Vanillylmandelic acid	$[M+H]^+$	$C_9H_{10}O_5$	Down	Up	ns
